# Pathways toward wearable and high-performance sensors based on hydrogels: toughening networks and conductive networks

**DOI:** 10.1093/nsr/nwad180

**Published:** 2023-06-22

**Authors:** Junbo Zhu, Jingchen Tao, Wei Yan, Weixing Song

**Affiliations:** Beijing Key Laboratory for Optical Materials and Photonic Devices, Department of Chemistry, Capital Normal University, Beijing 100048, China; Beijing Key Laboratory for Optical Materials and Photonic Devices, Department of Chemistry, Capital Normal University, Beijing 100048, China; Beijing Key Laboratory for Optical Materials and Photonic Devices, Department of Chemistry, Capital Normal University, Beijing 100048, China; Beijing Key Laboratory for Optical Materials and Photonic Devices, Department of Chemistry, Capital Normal University, Beijing 100048, China

**Keywords:** hydrogels, electronic skin, toughening networks, conductive networks, wearable sensors

## Abstract

Wearable hydrogel sensors provide a user-friendly option for wearable electronics and align well with the existing manufacturing strategy for connecting and communicating with large numbers of Internet of Things devices. This is attributed to their components and structures, which exhibit exceptional adaptability, scalability, bio-compatibility, and self-healing properties, reminiscent of human skin. This review focuses on the recent research on principal structural elements of wearable hydrogels: toughening networks and conductive networks, highlighting the strategies for enhancing mechanical and electrical properties. Wearable hydrogel sensors are categorized for an extensive exploration of their composition, mechanism, and design approach. This review provides a comprehensive understanding of wearable hydrogels and offers guidance for the design of components and structures in order to develop high-performance wearable hydrogel sensors.

## Teaser

This review summarizes the toughening and conductive networks of wearable hydrogels, highlighting representative enhancement strategies. It also discusses recent advancements and future development prospects for wearable hydrogel sensors.

## INTRODUCTION

The increasing demand for Internet of Things devices in healthcare applications is driving the exploration and development of wearable and flexible electronics [1]. Many electronic skins (E-skins) have been developed for applications in artificial intelligence, prosthetics, human-computer interaction, virtual reality, and health monitoring. Inspired by the human skin, the largest organ, E-skins possess softness, stretchability and self-healing properties and are equipped with sensors that can detect changes in environmental pressure, deformation, and temperature. Due to their similarities to human skin, such as flexibility, stretchability, bio-compatibility, and self-healing properties, hydrogels have garnered significant interest for use in electronic skin applications [2]. Hydrogels are three-dimensional (3D) networks of hydrophilic polymers that can absorb and retain large amounts of water. Their Young's modulus (500 kPa^−1^ MPa) is like that of human skin [3], which eliminates discomfort caused by rigid glass- or lithography board-based electronics. With high tensile rates, hydrogels can accurately detect large deformations of human joint motion, overcoming the limitations of low monitoring ranges seen in graphene [4,5], conductive sponges [6], and smart textiles [7]. Wearable electronic devices are prone to damage and deterioration over time, but self-healing hydrogels can repair themselves through non-covalent reactions such as *π–π* [8,9], hydrogen bonds [10–12], hydrophobic interactions [13–15], host-guest interactions [16], and ion coordination [17–19], as well as dynamic covalent reactions such as imine bonds [20], and boronic ester bonds [21], which have a great prospect of development. Figure S1 provides a summary of the developmental history of hydrogel sensors, and illustrates some typical examples of hydrogel sensor development at that time.

In the past two decades, the increasing focus on personal health and lifestyle has spurred extensive research dedicated to the development of hydrogel-based sensors. Figure S2 illustrates the significant growth in the number of publications and citations on hydrogel sensors in recent years. The total number of publications on hydrogel sensors has quadrupled, while citations have increased 5-fold from 2017 to 2022, signifying the growing traction and momentum of hydrogel research.

The 3D network of hydrogels imparts solid-like properties while also allowing for quick transport of substances due to the presence of aqueous phases. Toughened hydrogels including double-network (DN) [22], hydrophobic associated [9], and composite hydrogels [23], are reinforced through physical or chemical cross-linking, resulting in enhanced ductility and toughness. This makes them well-suited for integration into electronic devices that can conform to the self-supporting and repetitive stretching of human skin or joints. The introduction of electronic conductors or ions into hydrogels leads to the formation of conductive hydrogels (CHs). Commonly utilized conductive materials such as carbon-based materials [22], conductive polymers [24,25], and metal ions [26–29] enhance both conductivity and contribute to the formation of the matrix network structure. The hydrogel elastic matrix and conductive component are both vital elements in CHs.

**Figure 1.**
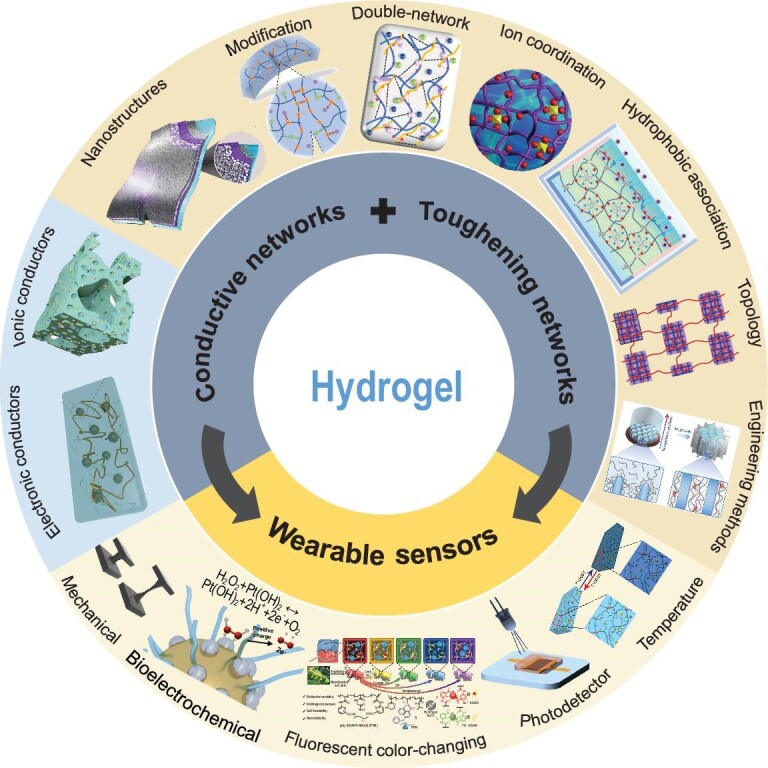
Schematic diagram of the enhancement of performance and the utilization of hydrogels. Adapted with permissions from refs [11,12,14,15,17–19,25–29].

Herein, we present a comprehensive discussion on the toughening and conductive networks of wearable hydrogels, highlighting various strategies and representative methods for enhancing their mechanical and electrical properties. These two properties will be critical for sensors with integrated performance in future applications, and our summary may serve as a valuable complement to previous reviews. Furthermore, an overview of recent advancements in wearable hydrogel sensors is presented, encompassing their composition, mechanisms, design strategies, and future development prospects (Fig. 1).

## TOUGHENING NETWORKS

The properties of hydrogel, including tensile strength, toughness, self-healing, adhesion, and anti-freezing, significantly impact in the sensing performance, stability, and lifespan of hydrogel sensors. Hydrogels possessing strength and toughness exhibit superior shape recovery, crack resistance, and fatigue resistance. The development of high-strength and tough hydrogels has become a focus of research. The main strategies for developing high-strength and tough hydrogels include: double-network [30], cross-linking [22], ion coordination [31], engineering methods [11], nanomaterial introduction [32], etc. Table S1 summarizes the experimental results of the mechanical properties of hydrogels produced by these various methods.

### Double-network

Traditional hydrogels, composed solely of a hydrophilic polymer such as polyacrylamide (PAM) hydrogels, have inadequate strength and toughness and do not meet the mechanical performance requirements of flexible wearable sensors. To address this, double-network hydrogels have emerged, comprising a brittle and rigid first network alongside a flexible and ductile second network. The first network is formed by physical interactions such as hydrogen bonding, cross-linking, and hydrophobic interactions, mainly serving a supportive function. The second network forms the primary gel matrix component, consisting of chemically bonded long chains of polymers. In double-network hydrogels, the toughening mechanism involves the dissipation of significant energy through cracking in the brittle first network. Meanwhile, the flexible second network ensures the hydrogel's integrity through stretching, enabling high water uptake and swelling.

Traditional pure chemically cross-linked hydrogels have poor shape recovery due to their non-reversible covalent bond, which negatively impacts their anti-fatigue performance and recyclability. To overcome this challenge, the concept of incorporating reversible dynamic bonds has been proposed. This encompasses hydrogen bonding [33], electrostatic interactions [34], hydrophobic association [34], and other physical cross-linking methods [25] for the creation of physical chemistry hybrid hydrogels or fully physically cross-linking hydrogels. Dynamic bonds breaking under external force can dissipate a large amount of energy and delay crack formation, thus enhancing the toughness of hydrogels. For instance, Sun *et al*. developed stretchable and conductive hydrogels via a hybrid double-network approach by combining rigid, physically cross-linked gelatin with tough, chemically cross-linked PAM and poly (3,4-ethylene dioxythiophene): poly (styrene sulfonate) (PEDOT: PSS) as a conducting component [35]. The double-network can be further interlocked by using physical entanglements and abundant dynamic hydrogen bonds, contributing to their improved mechanical properties and self-recovery abilities. The reversibility of these bonds also grants hydrogels the ability to rapidly recover and self-repair. For example, Yu *et al*. reported hydrophobicity-assisted multiple hydrogen bonding interactions among phenylalanine derivatives, increasing the elasticity and fatigue resistance of PAM hydrogel [36].

### Ion coordination

The ion coordination toughening strategy entails the incorporation of metal ions into the hydrogel matrix, forming coordination bonds with polymer chain groups like carboxyl groups. These ions act as physical cross-linking points within the network [31]. Li *et al.* utilized acrylic acid (AAC) and created DN Agar/AAC-Fe^3+^ physically cross-linked hydrogels by adjusting the amount of Fe^3+^ penetration [37]. The ion-coordination relationship of AAC-Fe^3+^ and the hydrogen-linked double helix of Agar can both be temporarily sacrificed during high deformation, allowing for faster energy release. However, the reverse AAC-Fe^3+^ contact can be regained after stiffness degradation, resulting in increased toughness. The remarkable mechanical properties of the hydrogen (elongation at break: 3174.3%) are derived from the multiple cross-linking and physical cross-linking through coordination between acids and groups on the molecular chain [37]. By employing ion coordination to fine-tune the interactions of hydrogel fillers, it becomes possible to enhance the mechanical properties of sensors. For instance, Li *et al*. achieved remarkable self-healing ability in the MXene PAA-Fe^3+^ hydrogel by facilitating a multitude of dynamic interactions between MXene nanosheets, PAA, and Fe^3+^, resulting in enhanced mechanical properties [32]. A comparison between the double-network and ion coordination in Table S1 reveals that the strength of DN hydrogel enhances upon the inclusion of an ion group. Consequently, by employing a well-designed coordination characteristics and dual-network structure, hydrogels can find applications across a wide range of scenarios.

### Hydrophobic association

Hydrophobic associative hydrogels can not only provide toughness to hydrogels but also exhibit exceptional mechanical, self-healing, and adhesion capabilities [38,39]. The hydrophobic domain is a physically cross-linked region where the hydrophobic groups on the polymer chain aggregate with each other in a water environment, resulting in the intramolecular or intermolecular association of the macromolecular chain. Hydrogels’ capability to dissipate external energy is enhanced by generating hydrophobic association domains that link two or more hydrophobic groups of polymer chains, serving as physical cross-linking points within the hydrogel network. The competition between the hydrogen bond formed by the solvent molecule and the polymer chain results in a decrease in the thickness of the hydrogel network chain and may even lead to the disruption of physical cross-linking. As a result, the hydrophobic region effectively prevents the hydrogel from undergoing excessive expansion [34].

Hydrophobic associative hydrogels can form micelles and facilitate the creation of polar microdomains along with bifunctional ionic surfactant. Adding emulsifiers or latex particles can stabilize the hydrophobic segments, further improving the mechanical properties of the hydrophobic hydrogels. For instance, xanthan gum, with its strong and highly branched structure, combined with sodium lauryl sulfate, can effectively stabilize the hydrophobic side of the hydrophobic hydrogel and enhance its strength and toughness [40]. However, hydrogen bonding between water molecules and the sensor surface can hinder the interaction between the sensor and the surface of the substrate and reduce or eliminate the hydrogel's adhesion in the case of underwater wearable hydrogel sensors. Although the hydrophobic effect can remove the hydration film from the hydrogel's surface and grant the sensor wet adhesion and self-healing abilities [41], hydrophobic hydrogels need to improve their adhesion while maintaining their hydrophobic microstructure.

### Topology

**Figure 2.**
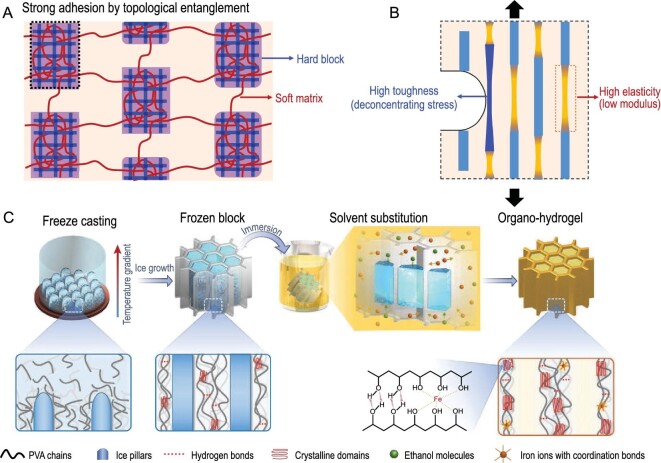
(A) A topological polymer network with hard blocks topologically entangled on a soft matrix. Adapted with permission from ref [25]. Copyright 2022 Springer Nature. (B) Mechanical principles of topological polymer networks with high toughness and elasticity. Adapted with permission from ref [25]. Copyright 2022 Springer Nature. (C) High toughness organic hydrogels obtained through freeze-casting. Adapted with permission from ref [11]. Copyright 2022 Wiley-VCH Verlag.

Due to the inherent plasticity of hydrogels, the design of topological structures can enhance their mechanical properties while also allowing for their utilization in unique scenarios. Management of the topological structure between polymer networks is a crucial method for enhancing the mechanical characteristics of hydrogels [42]. The topological microstructure refers to a cross-linked region that arises from the interaction between small molecules and large molecule chains, or between guest and host molecules. Liu and colleagues achieved the creation of a hydrogel resembling a tendon by strategically breaking the topological structure of a rigid block with a flexible substrate. This approach led to a hydrogel with a high elastic limit strain and increased strength capabilities (Fig. 2A) [25]. The hydrogel was developed based on the structure of the Achilles tendon, which contained parallel muscle bundles composed of interwoven collagen fibers in a proteoglycan matrix. Unlike a typical homogeneous interpenetrating polymer network, the topological polymer network in this hydrogel was heterogeneous, leading to stronger adhesion through topological entanglement. When the hard block broke, the stored elastic energy was released, causing the network to become stiffer and enhancing the hydrogel's elastic properties (Fig. 2B) [25]. The topology design can regulate both physical and chemical properties to customize hydrogels for different scenarios. For instance, hydrogels with a porous or highly branched network structure can offer a greater surface area and more binding sites for detecting analytes, leading to improved sensitivity and selectivity [43]. However, the current understanding of the relationship between different topological structures and hydrogel properties is limited, necessitating further research and exploration.

### Introduction of nanostructures

Incorporating nanostructures into hydrogels can adjust their physical and chemical properties, including porosity, mechanical strength, thermal stability, biocompatibility, and surface area. There are various types of nanofillers that can be introduced into hydrogels, including metallic nanoparticles [44], carbon-based nanoparticles [45], core-shell nanoparticles [46], silica nanoparticles [47], nanosheets [32], and nanofibers [48]. These fillers can be incorporated into the hydrogel network through a variety of methods, such as physical mixing [45] and electrostatic interactions [46].

The selection of nanofiller type for hydrogels depends on the intended application of the sensor. For example, incorporation of metallic nanoparticles can enhance the electrical conductivity of hydrogels, thereby making them well-suited for application in bio-sensors and electronic skin [44]. By incorporating specific response nanoparticles into hydrogels, they can exhibit responsiveness to various stimuli, such as temperature [45] and light [46]. Additionally, the introduction of fluorescent nanoparticles can render hydrogels capable of fluorescent color-changing properties [47]. The addition of carbon-based nanoparticles can augment the mechanical strength and toughness of hydrogels, thereby enhancing their suitability for load-bearing applications [45]. Nanofillers can also be employed to alter the surface properties of hydrogels. For example, the incorporation of silica nanoparticles can increase the surface area of the hydrogel, resulting in improved adsorption and release of drugs or other molecules [47].

Rigid nanosheets frequently act as physical cross-linking points and effective stress transfer centers within the network, thereby enhancing the toughness and ductility of hydrogels [32]. The arrangement of nanofibers during the strain process can effectively inhibit crack propagation [48]. These excellent mechanical properties are important to enhance the environmental tolerance and longevity. The structure of the self-healing nanofiber network is like the interwoven structure of repairable nanofibers found in human skin. This similarity gives the hydrogel an exceptionally high fatigue threshold of 2950 J m^−2^, and the sensor a remarkable strain sensing measurement factor of 66.8, setting a record [49].

### Functionalized modification

Hydrogels can be modified in various ways to upgrade their physicochemical properties for specific applications. These modifications can significantly enhance the selectivity, sensitivity, and stability of hydrogel sensors. An example of functionalization involves the utilization of carrageenan and Fe^3+^ to modify PAM through electrochemical methods. This process significantly enhances the mechanical strength, adhesion, and self-healing efficiency of hydrogels [50]. By introducing functional groups such as carboxyl [32] and amine [46] groups onto polymer chains, hydrogels can be utilized for the attachment of bio-molecules [51]. Ultraviolet-induced surface grafting is an effective strategy for increasing the toughness of hydrogels [52]. Under ultraviolet light, parts of the hydrogel network generate free radicals that trigger polymer polymerization. It plays the role of welding different networks and increasing the unity and mechanical properties of multiple groups of hydrogels [52].

### Engineering methods

Engineering methods can also be employed to tailor hydrogels with specific properties, and effective engineering approaches can optimize their performance. Various engineering techniques, such as freeze casting [11], electrospinning [48], and mechanical stretching [16], are employed to mimic the micro- and nano-anisotropic structures found in natural bio-materials, resulting in the production of hydrogels with high strength, toughness, tensile strength, and fatigue resistance. Dong *et al*. incorporated a frozen block of polyvinyl alcohol (PVA) in an iron chloride solution in ethanol (Fig. 2C) [11]. The dissolution of ice crystals in PVA confined within an ethanol solution gives rise to several characteristic phenomena, such as micro-gelatinization, the formation of stable, anisotropic honeycomb-like structures, the submicroscopic aggregation of hydrophobic amorphous PVA on the honeycomb walls, and the nanoscale ionic enhancement of PVA chains. The organic hydrogel possesses high strength (6.5 MPa), high tensile strength (1710% strain), and ultra-high toughness (58.9 MJ m^−3^) [11]. Hua *et al.* combined directed freezing and Hoffmeister effect techniques to modify the structure of PVA hydrogels on a multi-scale level, resulting in the combined strength and wear resistance of natural hamstrings [53]. Directed freezing can produce an anisotropic structure on a larger scale (micron-millimeter). Ni *et al*. were inspired by the soft membrane in the lobster belly. They utilized electrospinning technology to imitate its spiral structure, producing a nanofiber hydrogel with high strength (8.4 MPa) and a low fatigue threshold (770 J m^−2^) [48]. Engineering methods provide a powerful toolbox for the design and optimization of hydrogel sensors, enabling their integration into a wide range of applications. In order to make the application of hydrogel sensors a reality more quickly, it is important to develop a more advanced and convenient engineering technology.

## CONDUCTIVE NETWORKS

Hydrogel-based sensors necessitate components with suitable electrical conductivity. Since the hydrogel matrix itself is non-conductive, it becomes essential to introduce conductive materials into the hydrogel. These conductive fillers can be classified into two categories: ionic and electronic conductors, each of which will be discussed in detail below. The dispersibility, stability, and transmission of these conductive materials within the hydrogel matrix have a substantial impact on the sensing performance, stability, accuracy, and lifespan of hydrogel sensors. Table S2 presents a comparison of the conductivity of conductive hydrogels created from different combinations of substances.

### Electronic conductors

In electronic conductors, electrons possess a high degree of freedom and can flow through matter, creating an electric current. Common examples of electronic conductors incorporated into hydrogels include metal conductors, semiconductors, conductive polymers, and carbon materials.

**Figure 3.**
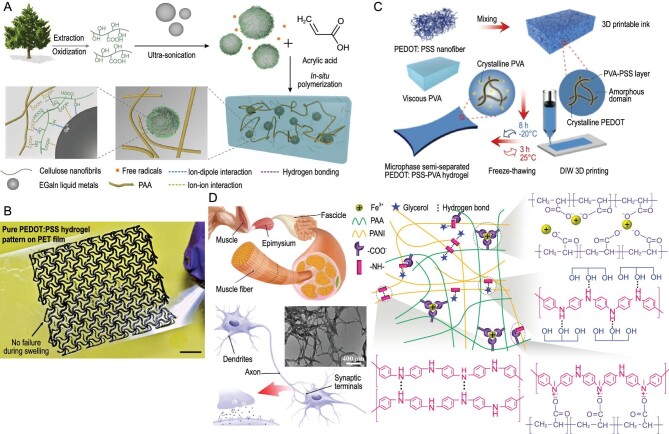
Electronic conductors which based CHs. (A) Schematic of the conceptual design and preparation process of PAA hydrogels encapsulated with CNFs-encapsulated LMNPs. Adapted with permission from ref [12]. Copyright 2022 Elsevier. (B) PEDOT:PSS Nanofiber Interconnection network hydrogel. Scale bar: 8mm. Adapted with permission from ref [24]. Copyright 2019 Springer Nature. (C) Principle and preparation of PEDOT:PSS-PVA conductive polymer hydrogel. Adapted with permission from ref [55]. Copyright 2022 Wiley-VCH Verlag. (D) Human muscle-inspired PANI-PAA hydrogel. Adapted with permission from ref [57]. Copyright 2020 American Chemical Society.

Gogurla *et al.* incorporated silver nanowire (AgNW) electrodes into polydimethylsiloxane elastomer films to facilitate carrier collection, and changes in resistance occurred upon stretching [26]. Liquid metals (LMs), which are alloys known for their high conductivity and fluidity, have garnered significant attention. The notable advantages of liquid metals include their high conductivity, excellent plasticity, and versatility in being shaped into various forms. However, the high cohesive energy of LMs presents challenges in reducing them to nanoparticles, and the weak interfacial cohesion results in macroscopic phase separation and inferior mechanical properties in conductive hydrogels based on LMs [38,12]. Combining it with the hydrogel strategy proves to be an effective method for addressing the problem. Recently, Ye and Jiang *et al*. used an interface engineering approach to alter the average diameter of LMs to liquid metals nanoparticles (LMNPs), ranging from 270 to 475 nm, by utilizing TO-Cellulose nanofibers (CNFs) as an interface stabilizer [12]. Polyacrylic acid (PAA)-CNFs-LMNPs hydrogels exhibited outstanding tensile characteristics (up to 2950%), solvent-independent conductivity (up to 0.45 S m^−1^), and high strain sensing sensitivity (GF = 12.5) [12]. The presence of CNFs at the interface prevented adjacent particles from coalescing. In addition, the plentiful surface polar hydroxyl or carboxyl groups on CNFs can engage in favorable interactions with the hydrophilic polymer matrix of hydrogels through hydrogen bonding (Fig. 3A) [12]. This interaction promotes energy dissipation and enhances the mechanical properties of hydrogels.

The electrical conduction mechanism of conductive polymers involves the transfer of electrons between conducting groups. They offer advantages such as good biocompatibility and processability, but their electrical conductivity and stability require improvement in hydrogel. PEDOT: PSS hydrogels are attracting significant attention due to their excellent biocompatibility with cells. The common method of preparation involves creating an interpenetrating polymer network (IPN) within a non-conductive gelatin template through stirring or *in situ* polymerization. Other synthetic methods, such as dry annealing and rehydration, can also be employed to create hydrogels with specific properties. Lu *et al.* successfully developed a pure PEDOT: PSS hydrogel with controlled isotropic or anisotropic expansion, achieved through mechanical constraint. This approach allowed for precise control of swelling characteristics and resulted in hydrogels with improved mechanical, electrical, and electrochemical stability [54]. This high-performance pure PEDOT: PSS hydrogel (Fig. 3B) utilizes an interconnected network of PEDOT: PSS nanofibers [24]. They developed a straightforward one-step synthesis approach, combining 3D printing and freeze-thaw methods, to create a unique semi-separation network using PEDOT: PSS nanofibers and PVA (Fig. 3C) [55]. The application of toughening methods enhances the mechanical properties of conductive polymers, making them suitable for use in sensors. Lee *et al*. achieved a uniform and conductive PEDOT: PSS gel with good ductility by effectively distributing PEDOT: PSS with PAM [56]. Xu *et al.* developed non-invasive glucose sensors for *in vivo* monitoring by constructing a composite hydrogel electrode using conductive PEDOT: PSS and Pb nanoparticles [9]. Like PEDOT: PSS, polyaniline and polydopamine (PDA) are well-known conductive polymers. Ge *et al.* incorporated polyaniline fibers into the PAA hydrogel network to develop a fiber-enhanced antifreeze self-healing hydrogel sensor. This design was inspired by the fiber-reinforced microstructure and mechanical conduction system found in human muscle (Fig. 3D) [57]. Conductive polymer fillers hold great promise for various applications. PDA, a substance secreted by Mytilus edulis, exhibits the potential to enhance the hydrophilicity of hydrophobic substrates. Polyaniline and PDA offer similar conductivity to PEDOT: PSS but at a lower cost. However, they exhibit weaker stability in water and lower solubility. To address these limitations, nanoparticles or additives that promote solubility can be incorporated into the system.

Carbon-based materials, such as graphene and carbon nanotubes (CNTs), offer a remarkable capability to enhance the conductivity of hydrogels [45,58]. They increase the effective contact area within hydrogels and allow for customized adjustment of their conductivity.

**Figure 4.**
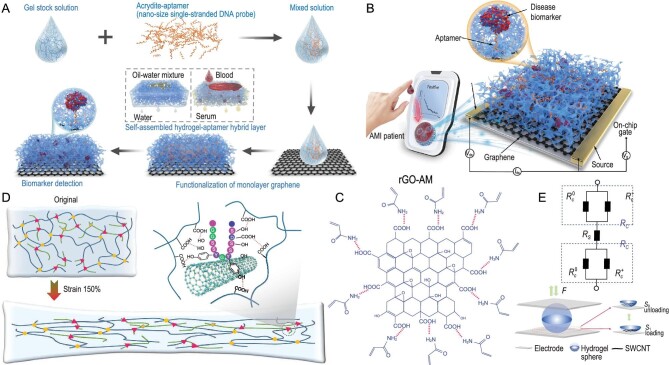
Carbon based CHs E-skin. (A) 3D-schematic diagram of the manufacturing process of the bio-sensor. Adapted with permission from ref [60]. Copyright 2022 Elsevier. (B) Schematic diagram of the prepared 3D bio-sensor with H-AHL structure. Adapted with permission from ref [60]. Copyright 2022 Elsevier. (C) Synthetic hydrogels with vinyl-modified rGO and acrylate monomers at the edges. Adapted with permission from ref [62]. Copyright 2019 Wiley-VCH Verlag. (D) The nano-structure of XSBR/SSCNT sensor before and after tensile force. Adapted with permission from ref [63]. Copyright 2022 Wiley-VCH Verlag. (E) The SWCNT/SA-based hydrogel ball sensor. Adapted with permission from ref [64]. Copyright 2015 Royal Society of Chemistry.

Graphene has high electrical and thermal conductivities, zero effective mass, and desirable mechanical properties [9,59]. Yet the problem of nonspecific adsorption of non-target molecules on the graphene surface needs to be solved. Wang *et al*. overcame this limitation by integrating a hydrogel layer and an adapter layer to modify the graphene conductive channels. The resulting hydrogel-adapter hybrid layer (H-AHL) structure was combined with a 3D bio-sensor that self-assembled through a graphene field-effect transistor (Fig. 4A) [60]. The aptamers in the H-AHL structure can specifically interact with the target biomarkers, while the hydrogel can filter out nonspecific molecules in whole blood. This leads to efficient and comprehensive screening of acute myocardial infarction biomarkers in patient samples (Fig. 4B) [60]. The presence of epoxy, hydroxyl, and carboxyl groups on the surface and edges of reduced graphene oxide (rGO) facilitates easy modification, making it an ideal connecting center for the formation of polymeric composites [61]. According to Yue *et al.*, free radical polymerization was employed to synthesize hydrogels with a conductivity of 27.2 S m^−1^, utilizing modified rGO and acrylate monomers that incorporated abundant ionic groups (Fig. 4C) [62]. The high cost associated with traditional graphene fabrication processes hinders its widespread application in sensor preparation. We employed a laser-induced method to fabricate a three-dimensional porous graphene network on a polymer film, which not only has low cost but also exhibits excellent dispersibility. This property makes it highly suitable for dispersion and electrode preparation in hydrogels [4].

Lin *et al*. achieved an even dispersion of CNTs without the use of additional artificial additives by modifying them with hydrophilic sericin (SS) and carboxyl styrene butadiene rubber (XSBR) [63]. The integrated properties of the XSBR/SSCNT sensor were achieved due to the formation of a hydrogen-bonded crosslink network between the sericin-modified CNTs and the polar carboxyl group of the XSBR. The nano-structure of the XSBR/SSCNT sensor was schematically depicted before and after the application of tensile force (Fig. 4D) [63]. During the stretching process, the interfacial hydrogen bonds between SSCNT and XSBR promoted the orientation of chain segments and resulted in strain-induced self-reinforcement. Besides, the fabrication of complicated microstructures and nanostructures need high costs. Starting from the changes in material properties and contact geometry, Tai *et al.* developed a simply designed, low-cost bio-based sensors. The sensor was a conductive single-walled carbon nanotube (SWCNT)/sodium alginate hydrogel ball with a ball-electrode contact mode wherein the CNTs enhanced its sensitivity (Fig. 4E) [64].

However, achieving high conductivity in hydrogels filled with electronic conductors such as nanowires, CNTs, and graphene can be challenging due to factors such as the random distribution of nanofillers, gaps between them, or insufficient contact between the nanoconductors. Moreover, increasing the concentration of conductive fillers may adversely affect the mechanical or functional properties of the hydrogel at low concentrations [63]. Therefore, achieving an even distribution of carbon-based material throughout the hydrogel network is crucial. These materials are more hydrophilic compared to other fillers, and uneven distribution could have a negative impact on the stability and reactivity of the sensor. Enhancing the dispersion of carbon-based materials within the hydrogel matrix can be achieved through the utilization of surface modifications or functionalization techniques. This can improve the compatibility between the hydrophilic carbon-based materials and the hydrogel matrix, leading to a more uniform distribution. Additionally, the use of suitable mixing methods, such as sonication or high-shear mixing, can aid in achieving better dispersion. Furthermore, careful control of the concentration and loading of carbon-based materials is essential to maintain the desired conductivity while minimizing any adverse effects on the mechanical or functional properties of the hydrogel.

### Ionic conductors

The way ions transport through the hydrogel network is comparable to the movement of ions within the human body [65]. Three common types of ionic conductors are commonly employed to enhance the electrical properties of hydrogels: electrolytes, ionic liquids, and ionic polymers.

An effective and widely used approach to create conductive hydrogels involves the incorporation of electrolyte solutions because of their low cost and high ionic conductivity. These solutions typically contain metal cations such as Na^+^ [39,66], Ca^2+^ [23], Fe^3+^ [67], and NH_4_^+^ [68]. When the hydrogel is immersed in an electrolyte solution, the ionic groups present in the electrolyte solution penetrate the hydrogel and undergo an ion exchange reaction. This process leads to an increase in the ionic conductivity of the hydrogel. However, the stability of the electrolyte-based conductive hydrogels is often compromised as they are susceptible to environmental influences. Additionally, the ions tend to aggregate on the electrode surface, leading to a reduced lifespan. Moreover, ion leakage and imbalanced distribution can negatively impact the mechanical and electrical properties of hydrogels. Despite these challenges, the concentration distribution resulting from ion movement can still elicit an electrical response. Di *et al.* incorporated sodium chloride into PVA hydrogel to enhance its conductivity. They further manipulated the structure, mechanical properties, and sensitivity by adjusting the crystallization time and *in-situ* soaking time. Additionally, the entanglement of the polymer chain network was influenced by the hydrogen bond network and salting-out effect [66]. Adequate mechanical properties are essential to support good electrical conductivity.

**Figure 5.**
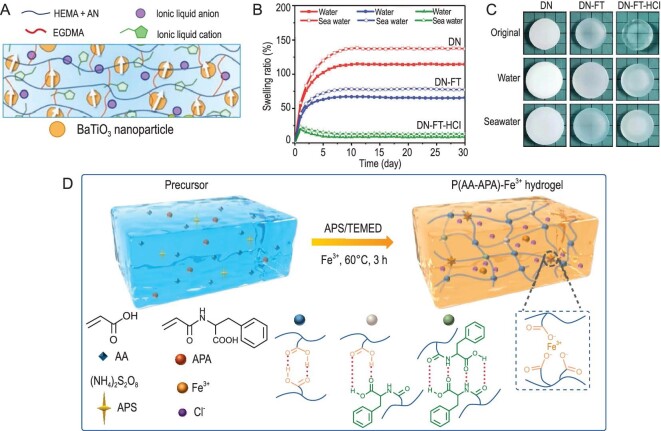
Ion based CHs. (A) Composite hydrogels made from piezoelectric nanoparticles BaTiO_3_ combined with ionic liquids. Adapted with permission from ref [70]. Copyright 2019 American Chemical Society. (B) Swelling kinetics of DN, DN-FT and DN-FT-HCl hydrogels in water and artificial seawater. Adapted with permission from ref [73]. Copyright 2021 Wiley-VCH Verlag. (C) Images of DN, DN-FT and DN-FT-HCl hydrogels after 30 days of swelling in water and artificial seawater. Adapted with permission from ref [73]. Copyright 2021 Wiley-VCH Verlag. (D) Schematic illustration of the preparation of the P (AA-APA)-Fe^3+^ hydrogels. Adapted with permission from ref [74]. Copyright 2023 Elsevier.

Ionic liquids, also known as molten salts, exhibit strong conductivity, thermal stability, low volatility, and chemical stability at room temperature [69]. Similarly, when the hydrogel is immersed in an ionic liquid, it undergoes an ion exchange reaction, leading to an increase in the ionic conductivity of the hydrogel. The combination of ionic liquids and nanomaterials can result in unique properties. In composite hydrogel systems formed by mixing ionic liquids with piezoelectric nanoparticles like BaTiO_3_, a distinct electrical response to low-frequency tactile stimuli can be observed (Fig. 5A) [70]. The combination of these two piezoelectric effects results in an output voltage of up to 8 mV and exhibits an anisotropic response. Ionic liquids continue to encounter challenges related to high costs, partial toxicity, and electrostatic interference. We believe that the development of deep eutectic systems can provide further advancements in conjunction with ionic liquids. In addition, zwitterions enhance the transmission of ionic signals through electrostatic interaction between electrolyte ions and amphoteric ion groups, while also retaining the advantageous properties of ionic liquids [71]. Due to the nature of zwitterions, the cations and anions of the electrolyte can be easily separated without requiring a strong electrostatic attraction during ion migration. They can readily transfer onto the electrode surface [72]. In addition, the charged groups of amphoteric hydrogels exhibit a strong electrostatic interaction with water molecules, resulting in a high degree of hydration for these charged groups. Ion migration channels are formed between hydration layers of polyzwitterion chains, which significantly improve the efficiency of electrolyte transfer. The anti-swelling properties of DN-FT-HCl hydrogels, developed by Ren *et al*. were attributed to the protonation of amphoteric ionic polymers (Fig. 5B) [73]. These hydrogels can be utilized for underwater motion detection (Fig. 5C) [73].

Ionic polymers have excellent ionic conductivity, chemical stability, favorable mechanical and processing properties, making them highly suitable for fabricating complex sensors and devices. The ionic conductivity of hydrogels can be enhanced through the ion exchange reaction between the hydrogel and ionic polymers. In comparison to the two methods, achieving high ionic conductivity with ionic polymers requires the introduction of a significant number of ionic groups. However, it should be noted that certain ionic polymers may be prone to dissolution or degradation, which can adversely affect their stability and lifespan. Recently, Shen *et al.* reported a hydrogel ionic skin prepared by one-step free radical polymerization of *n*-acryloyl Phenylalanine (APA) and acrylic acid (AA) in ferric chloride solution (Fig. 5D) [74]. APA is utilized to facilitate the formation of multiple hydrogen bond interactions. The coordination interaction between Fe^3+^ and carboxyl groups imparts adjustable mechanical properties to the hydrogels, including high toughness, excellent elasticity, and fatigue resistance. The ionic skin had a reliable signal output for both large movements, such as wrist flexion and walking, as well as delicate movements, such as speech and eye rotation [74]. However, the ion-gel exhibits high transfer resistance and a low diffusion coefficient due to its polymer structure, which hinders ion mobility. Researchers are still in the process of exploring the interaction mechanism between ion-based conductors and hydrogels, as well as the relationship between the structure and properties of ion-based conductors. Additionally, there is a need to develop more innovative hydrogels inspired by various organisms.

The toughening network and conductive network are crucial for the performance of hydrogel sensors. In the field of stretchable electronics, it is vital to achieve high levels of stretchability, while maintaining electrical performance and reliability [75]. The toughening network directly influences the sensor's stretchability, resilience, fatigue resistance, shear resistance, adhesion, self-healing capabilities, environmental tolerance, and sensing specificity. On the other hand, the conductive network enables hydrogels to convert various stimuli into detectable electrical signals. Different conductive mechanisms and materials have a direct impact on the sensor's sensing speed, accuracy, and stability. Therefore, the toughening network and conductive network are the two key aspects to consider when developing and designing hydrogel sensors with exceptional sensing capabilities.

The toughening network, conductive network, and sensor are interdependent and synergistic in nature. The structural design and interplay of the toughening network have a direct impact on the transport dynamics of ions or electrons within the conductive network. The performance of a toughening network, including deformation recovery, shear resistance, and self-healing, is closely linked to factors such as the concentration gradient of conductive materials, reversible transmission, and even the reconstruction of the conductive network. These aspects directly impact the overall performance of the sensor. The ion coordination-based toughening network not only facilitates the formation of a microphase separation structure for energy dissipation but also imparts electrical conductivity to the hydrogel. Additionally, the incorporation of filling materials not only establishes the conductive network within the hydrogel but also alters its mechanical properties. Achieving a balance between the mechanical and electrical properties of hydrogels is crucial for attaining excellent sensing performance.

## SENSORS

Hydrogels are extensively employed in the development of soft, smart, and wearable sensors owing to their unique attributes of flexibility, stretchability, toughness, self-healing, adhesion, and biocompatibility. These sensors play a pivotal role in monitoring human movement signals, diseases, and environmental conditions, driving us toward a healthier, more informed, and intelligent era. The mechanical properties, electronic properties, and functional design of hydrogels are instrumental in achieving these goals. The diverse applications of hydrogels as sensors can be categorized based on their sensing principles. These include their utilization as mechanical sensors [76], electrochemical bio-sensors [10,27], fluorescent color sensors [77], photoelectric sensors [78], and temperature sensors [79]. Each of these applications highlights the versatility of hydrogels in various sensing domains. A comprehensive list of hydrogel-based sensors can be found in Table S3.

The design of sensors plays a crucial role in their functionality. Initially, researchers achieved stretchability with low stiffness by implementing various deformable designs, such as a buckled structure in a neutral mechanical plane or a rigid active cell island with wavy interconnects [80]. In current hydrogel sensor designs, there is a focus on incorporating functional fillers or cross-linking agents directly onto the hydrogel substrate. This approach aims to improve the bio-compatibility of the sensor and is crucial for achieving outstanding mechanical and electrical properties of the hydrogel substrate. The thickness and size of sensors also play a crucial role in their performance. With the trend toward miniaturization, sensors are being developed on a micro-scale, aiming for portability and user-friendly capabilities. If the sensors are too thick, they may hinder skin and air contact, increasing the risk of infection. Therefore, achieving optimal thickness is essential, requiring a meticulous preparation process. Furthermore, in an era of rapid intelligence development, integrating hydrogel sensors with smart devices is a key challenge.

In future hydrogel sensor designs, it will be crucial to perform qualitative and quantitative analyses of both the mechanical and conductive properties. If the mechanical and conductive properties are inappropriate, the sensitivity of the sensor will be greatly reduced. Excessive mechanical properties can result in slow response to external stimuli and low sensitivity, while high conductive properties can be easily affected by the environment, leading to poor stability and low sensitivity. Therefore, it is essential to strike a balance between the mechanical and conductive properties to achieve optimal sensitivity in hydrogel sensors. Similarly, an increase in the elastic modulus corresponds to higher stiffness, but it also results in a weaker response to external stimuli and lower sensitivity. These principles apply to all of the sensors described below, which, despite their different sensing mechanisms, aim to possess similar integrated electrical and mechanical properties.

### Mechanical sensors

Mechanical sensors operate on different physical principles, such as piezoresistance, piezoelectricity, and capacitive sensing, to detect changes in the physical properties of their environment. Piezoresistive materials alter their electrical resistance when subjected to mechanical stress or strain, while piezoelectric materials generate an electrical charge in response to mechanical stress or strain [81]. Capacitive sensors use two conductive surfaces separated by a dielectric material; the capacitance changes when an applied force or pressure alters the distance between the conductive surfaces. These changes can be measured to determine the applied force, pressure, or displacement. For instance, of a capacitive sensor, the capacitance is related to the area of the electrode, the distance among electrodes, the thickness of the hydrogel, and the dielectric constant of the hydrogel.


\begin{eqnarray*} {\rm{C}} = \varepsilon {\rm{A}}/{\rm{d}} \end{eqnarray*}

The relationship between capacitance (C) and dielectric constant (${\varepsilon}$), electrode area (A), and electrode spacing (d) is shown above. The capacitive sensor consists of two parallel plates, typically made of conductive materials as the electrodes, coated with a thin layer of hydrogel as the dielectric material separating the plates. The capacitance of the sensor is proportional to the dielectric constant of the hydrogel layer and the surface area of the plates. When a hydrogel-based capacitive sensor is exposed to a change in its environment (e.g. humidity, temperature, pH, pressure), the water content in the hydrogel changes, leading to a corresponding change in its dielectric constant and capacitance. A fully repaired capacitive strain sensor with a high tensile rate of 1080% was recently built by Jiang *et al*., using a dual-dynamic network technique [45]. Strong tolerance and quick healing for severe knotting and distortion were provided by combining the conductive nanowire assembly with a dual dynamic network based on *π–π* attraction and Ag-S coordinated bonding. Additionally, achieving a perfect fit between the sensor and non-flat human skin poses a challenge in accurately detecting human micro-motions, such as pulse beats and changes in facial expression [80].

**Figure 6.**
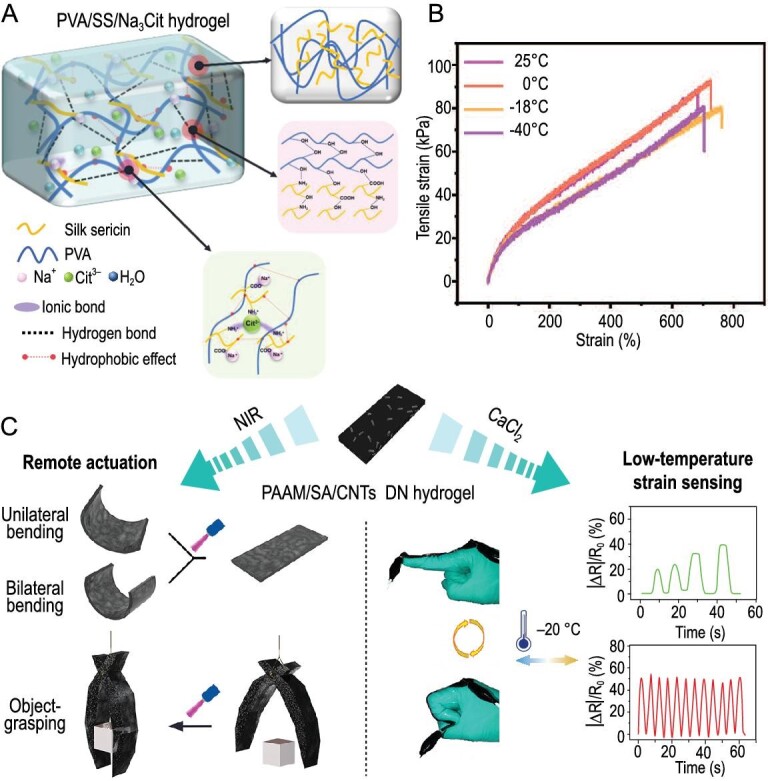
Strain and pressure hydrogel sensors. (A) Ionic conductive hydrogels constructed from SS with rich binding sites. Adapted with permission from ref [17]. Copyright 2022 American Chemical Society. (B) Tensile stress-strain curve of the gel after cold storage at different temperature gradients. Adapted with permission from ref [82]. Copyright 2022 Wiley-VCH Verlag. (C) DN hydrogel for applications in remote actuation and low-temperature strain sensors which are based on PAM/SA/CNTs. Adapted with permission from ref [83]. Copyright 2021 American Chemical Society.

For wearable hydrogel electronic skin to accurately detect human movement and physiological signals, it is crucial to consider the following factors pertaining to the hydrogel sensor: mechanical strength, tensile force detection range, minimum detection limit, self-healing properties, biocompatibility, and adhesion. Wang *et al.* created a composite hydrogel (PVA/SS/Na_3_Cit) employing SS (–COO–, –NH_2_, and –OH) with a lot of binding sites (Fig. 6A) [17]. This hydrogel exhibits numerous physical interactions, such as hydrogen bonds, ionic coordination, and hydrophobic interactions, contributing to its high tensile strength (4.42 ± 0.32 MPa) and elastic modulus (3.14 ± 0.26 MPa). The hydrogel displayed excellent ionic conductivity, a broad operational range, high sensitivity, and stability due to the coordinating effects of Na^+^ and Cit^3−^ ions. Human motion can be instantly recognized in any direction based on the change in relative resistance. After identifying the relaxation phase of the human body's significant deformation work, the self-healing property can swiftly return. To increase self-adhesion, prevent ice formation, and reduce water loss, Sun *et al.* added basic water-glycol (EG) to organic hydrogels. The capacity of the organic hydrogels to perform in harsh environments was enabled by the formation of numerous intermolecular hydrogen bonds between EG and water molecules (Fig. 6B) [82].

The mechanical hydrogel sensor also requires robust anti-interference capability and stability to minimize the influence of external factors on the detection results. As reported by Zhang *et al.*, a PAM/sodium alginate/carbon nanotube (PAM/SA/CNTs) double-network hydrogel demonstrated remarkable strength and toughness. The incorporation of CNTs into the PAM/SA DN hydrogel network enabled the development of hydrogels with adjustable photomechanical deformations, such as object gripping and bending (Fig. 6C) [83]. The hydrogel strain sensor exhibited good low temperature sensing capability after additional CaCl_2_ addition. To explore hydrogels with higher sensitivity, it is crucial to conduct further research on various mechanical sensing materials and explore different combination approaches.

### Electrochemical bio-sensors

The sensing mechanism of electrochemical bio-sensors relies on the electrochemical properties of the sensing elements and their interactions with the analytes. When the analyte interacts with the sensing elements, it has the potential to initiate or catalyze an electrochemical reaction. This reaction can be detected using various electrochemical techniques, including amperometry, potentiometry, or voltammetry. To enhance their electrochemical properties and sensitivity, electrochemical bio-sensors employ a variety of sensing elements and transducer surfaces, commonly known as ‘signal amplification strategies’. Electrochemical bio-sensors have been widely applied in various fields, including clinical diagnosis, environmental monitoring, and food safety.

Electrochemical sensors employ electrochemical reactions for analyte detection. To ensure their stability, durability, and convenience, the sensors require an appropriate substrate with suitable materials and dimensions. The mechanical and electronic properties of hydrogel significantly affect the accuracy of the sensor's signal. Electrochemical sensors are suitable for detecting bio-molecules, ion concentrations, and gaseous substances. For example, Das *et al.* synthesized a novel phenazine-based sensory receptor by multiple inter- and intramolecular carbon nitrogen bond fusions. This receptor was utilized for the detection of ammonia as well as various levels of aliphatic amines (primary, secondary, or tertiary) [84]. In the field of supramolecular chemistry, Bej *et al.* developed an MOF-based hydrogel membrane to detect formaldehyde gas phase using two kinds of luminescent porous networks, offering a novel application for supramolecular hydrogel technology [85].

**Figure 7.**
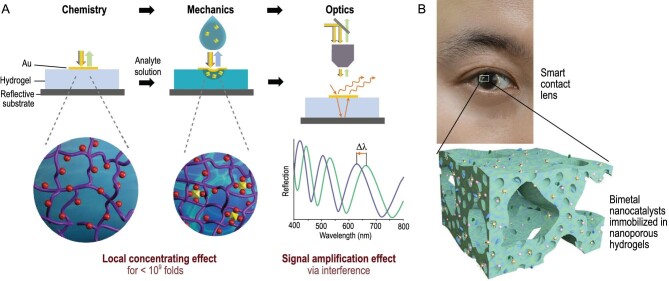
Hydrogel electrochemical bio-sensors. (A) Complete chemical-mechanical-optical signal transduction process of hydrogel interferometer platform. Adapted with permission from ref [18]. Copyright 2018 Wiley-VCH Verlag. (B) Schematic of GMCC preparation and color change of glucose sensor. Adapted with permission from ref [88]. Copyright 2022 Wiley-VCH Verlag.

The creation of microdevices for exceptionally accurate molecular detection is crucial given the increasing demand for environmental and health monitoring [86]. To enable precise electrochemical monitoring of environment-specific chemicals, the 3D network of hydrogels can accommodate a large quantity of ligands specifically designed for analytes. Sun *et al*. introduced a simple yet versatile sensing platform utilizing hydrogel interferometers. Ligands are integrated into the hydrogel networks, leading to a local enrichment effect when the analyte binds to the ligand. Subsequently, the optical signal is amplified, enhancing the overall detection capabilities (Fig. 7A) [18]. Monitoring the 24-hour urine copper excretion, with levels exceeding 100 *μ*g per 24 hours, can aid in the diagnosis and management of Wilson's disease [18]. In a recent study conducted by Das *et al.*, they developed the first azophenine-based chemical sensor capable of non-invasive diagnostic monitoring of Wilson's disease [87]. Azophenine and its various cyclized and uncyclized organic derivatives, as well as coordination complexes, exhibit exceptional redox properties. This sensor enables the measurement of exchangeable copper levels in serum and urine samples that aids in the detection and assessment of Wilson's disease [87].

The advantages of non-invasive glucose monitoring for individuals with diabetes are increasingly being acknowledged by the general population. Kim *et al*. developed a smart contact lens using hydrogel for the purpose of monitoring glucose levels in tears. To achieve this, they immobilized gold-platinum bimetallic nanocatalysts within nanoporous hydrogels (Fig. 7B) [88]. These nanocatalysts facilitate the rapid decomposition of hydrogen peroxide generated by the redox reaction of glucose oxidase and nanoparticle-mediated charge transfer. The glucose levels measured by the contact lens were consistent with those measured by a glucometer, demonstrating the potential for continuous, rapid, and non-invasive detection of blood glucose levels.

### Fluorescent color sensors

Fluorescent color-changing hydrogel sensors detect target analytes through the fluorescence properties of the sensing molecules, the volume changes of the hydrogel, and the specific interactions between the sensing molecules and the target analyte. Typically, the sensing molecules are integrated into the hydrogel matrix, which serves as a scaffold to keep them in place. When illuminated by light of a specific wavelength, the sensing molecules become excited and emit light at distinct wavelengths. When these sensing molecules come into contact with the target analyte, their fluorescence properties undergo changes, resulting in alterations in the intensity or wavelength of the emitted light. The swelling or shrinking of the hydrogel can modify the spacing between the sensing molecules, consequently influencing their fluorescence properties. This change in the hydrogel's dimensions is brought about by its interaction with the target analyte. The fluorescent color-changing hydrogel sensors find wide-ranging applications in environmental monitoring, food safety, and bio-medical engineering.

**Figure 8.**
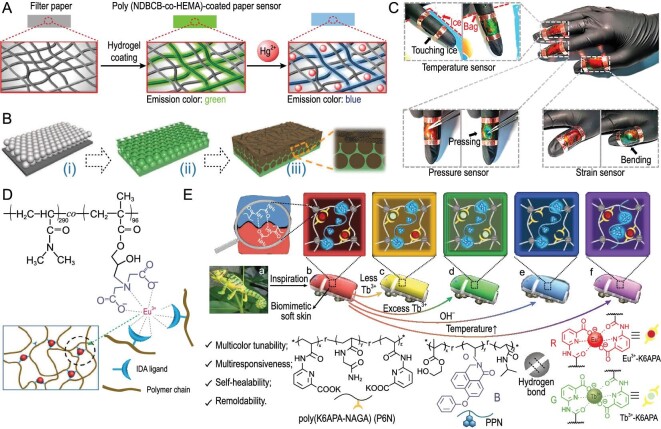
Fluorescent hydrogel sensors. (A) Preparation and visual detection schematic diagram of Hg^2+^ chemical sensor based on fluorescent hydrogel-coated paper. Adapted with permission from ref [7]. Copyright 2018 Wiley-VCH Verlag. (B) The formation process and microstructure diagram of color film with polymer-conductive hydrogel hybrid structure. (i) Silica colloidal crystal template prepared by vertical deposition method. (ii) PU inverse opal scaffold membrane. (iii) Fill CNTs-PDA conductive hydrogel. Adapted with permission from ref [89]. Copyright 2020 Wiley-VCH Verlag. (C) Optical photos of conductive fiber liquid crystal hydrogels pasted on different fingers to detect temperature, pressure and tension, respectively. Adapted with permission from ref [91]. Copyright 2018 PNAS. (D) Chemical structure of poly (*N,N*-dimethylacrylamide-co-glycidyl methacrylate) copolymer and Eu^3+^ ion. Adapted with permission from ref [90]. Copyright 2018 Wiley-VCH Verlag. (E) Schematic illustration showing the material design of the multicolor fluorescent polymer hydrogels, as well as the developed bio-mimetic soft skins with the adaptive color-changing behavior. Adapted with permission from ref [19]. Copyright 2022 Wiley-VCH Verlag.

The sensitivity of fluorescent sensors is also influenced by the mechanical and electrical properties inherent in the fluorescent hydrogels. Specifically, the high conductivity of fluorescent molecules can result in a shorter excitation lifetime, highlighting the need for meticulous design considerations. Zhang *et al.* have successfully developed a flexible paper/textile film chemical sensor that is specifically sensitive to Hg^2+^ [7]. This sensor overcomes the limitation of conventional Hg^2+^ sensors, which are often insensitive, by utilizing a hydrophobic, dense, and rigid material that effectively prevents the flow of aqueous solutions. (Fig. 8A) [7]. The grafted thiourea molecule reacts chemically with Hg^2+^, resulting in a noticeable color transition of the hydrogel from green to blue. The porous structure of the paper/textile fiber stack promoted Hg^2+^ diffusion into the hydrophilic hydrogel matrix, enabling detection of Hg^2+^ as small as a nanometer in aqueous solutions.

There are three common methods for producing color-changing polymer compounds. The first method involves incorporating chromophores that alter their structure or state in response to stimuli [7]. The second method is creating ‘structural’ color through manipulation of ordered nanostructures to influence photon propagation [89]. The third method revolves around generating intense luminescence using metal centers of luminous lanthanide metals (such as Eu and Tb) that exhibit dynamic coordination with polymers [90].

For example, Wang *et al.* developed a hybrid color film inspired by chameleons’ color-changing ability and mussels’ adhesion. The capacity of the chameleon to quickly change color depends on the presence of guanine nanocrystalline lattice structures that are not densely packed. The color film was fabricated by filling a polyurethane (PU) inverse opal scaffold with a carbon nanotube PDA hydrogel (Fig. 8B) [89]. The film acquired reliable tensile properties due to the presence of the PU layer, while the reverse opal structure contributed to the vibrant structural color exhibited by the film. The film's color can be modified in response to various stimuli, including temperature, pressure, and tension, by manipulating the volume or internal nanostructure of the composite liquid crystal hydrogels composed of hydroxypropyl cellulose (HPC), poly (acrylamide-co-acrylic acid) (PACA), and CNTs (Fig. 8C) [91]. Weng *et al.* used a hydrophilic poly (*N,N*-dimethylacrylamide) matrix to create a tunable luminous hydrogel through iminodiacetate (IDA) coordination to europium (Fig. 8D) [90].

The dynamic Lanthanide complexes in the hydrogel allow for switchable luminescence and sol-gel transitions in response to stimuli such as pH, temperature, metal ions, sound waves, and forces, due to their reversible synthesis and dissociation [19]. Liu *et al.* recently assembled a supramolecular polymer network by arranging different fluorophores, such as blue (B) aggregation-induced emission fluorophores and red/green (R/G) lanthanide coordination fluorophores, into various polymer chains (Fig. 8E) [19]. The fluorescence intensity of each of the B and R/G fluorophores could be separately modulated by various external stimuli, resulting in a multicolor fluorescence response with multiple responses. Moreover, inkjet printing technology can facilitate the practical application of these luminescent materials, paving the way for the development of commercially viable products in the near future [92,93]. This encompasses supramolecular materials with a wide array of potential societal applications.

### Hydrogel photodetectors

**Figure 9.**
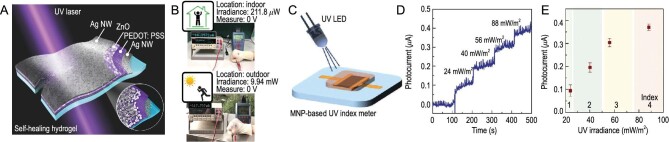
Hydrogel-based optoelectronic E-skin. (A) Schematic diagram of self-powered, self-healing and wearable UV photodetectors. (B) A light-sensing device attached to a human hand is used to measure ultraviolet radiation in a simulated indoor and outdoor environment. (A and B) Adapted with permission from ref [28]. Copyright 2020 American Chemical Society. (C) The schematic diagram of the UV sensor. (D) Photocurrent measured under different UV irradiation power. (E) The relationship between photocurrent and UV irradiance power. (C–E) Adapted with permission from ref [26]. Copyright 2020 Wiley-VCH Verlag.

Hydrogel photodetectors are devices that utilize hydrogel materials to detect and convert light into electrical signals. Light incident on the hydrogel material induces changes in its optical properties, such as absorption or scattering. These changes are then transduced into electrical signals through various mechanisms, such as photoconductive [23], or photoelectric effects [28]. Hydrogel photodetectors have a wide range of applications in bio-medical imaging, environmental monitoring, and optical computing. Tsai *et al.* reported a self-powered, self-healing, and wearable UV photodetector created by integrating agarose and PVA double-network hydrogel (Fig. 9A) [28]. The photodetector utilized AgNWs as both the top and bottom electrodes, while the photoactive layers consisted of a combination of PEDOT: PSS and ZnO nanocomposites. Under high illumination intensities, the Coulomb's interaction between high-density photoexcited carriers manifest themselves in reducing the internal electric field built at PEDOT: PSS/ZnO interface. As a result, the carriers were recombined before being collected by the electrodes, resulting in photocurrent generation. The UV viability of this ‘E-skin’ was demonstrated through simulations of both indoor and outdoor UV tests (Fig. 9B) [28].

Doping special functional materials presents another strategy for controlling the photoelectric properties of hydrogels. By incorporating these materials, the mechanical and electrical properties of hydrogels can be enhanced, while simultaneously allowing for the adjustment of light response and photoelectric characteristics. Gogurla *et al.* created an artificial photoelectric skin by dispersing melanin nanoparticles (MNPs) in a protein hydrogel-elastomer hybrid (Fig. 9C) [26]. This hybrid material was subsequently utilized for applications in a dopamine sensor and a UV index meter, demonstrating the versatility of the artificial photoelectric skin. As *n*-type semiconductors, MNPs generated more charge carriers when illuminated, which made the orbital hybridization hydrogel photoelectrically sensitive (Fig. 9D and E) [26]. The artificial photoelectric skin interacted with both the small molecules in the environment and the light passing through the transparent silk protein hydrogel window. Ding *et al*. achieved the development of a transparent and optically anisotropic hydrogel by embedding a two-dimensional paramagnetic material, specifically cobalt-doped titanium oxide, into a polymer matrix [94].

Despite their advantages, hydrogel photodetectors still face challenges related to sensitivity and signal-to-noise ratio. Enhancing the detection sensitivity and minimizing noise sources, such as dark current or interference, are critical aspects that need to be addressed in order to improve the overall performance and reliability of hydrogel photodetectors. Hydrogel photodetectors hold potential for integration with other functionalities, such as energy harvesting or biosensing capabilities, creating multifunctional devices with enhanced versatility and utility. These devices can enable self-powered sensing systems or facilitate simultaneous detection of multiple analytes in complex biological or environmental samples.

### Temperature sensors

**Figure 10.**
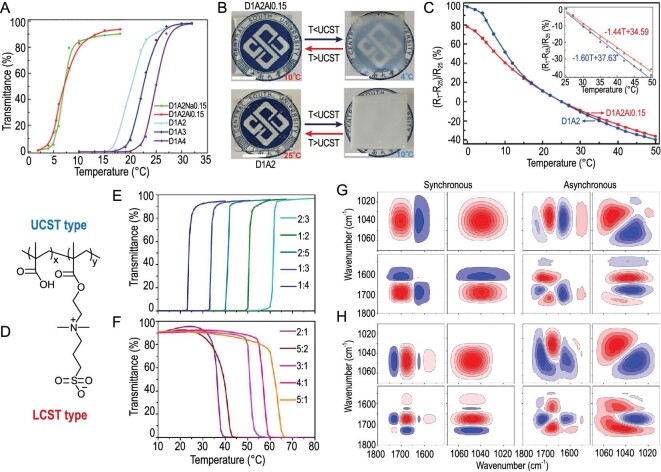
Hydrogel temperature sensors. (A) The relationship between transparency of UCST hydrogel (D_x_A_y_M_z_) and temperature. Here, x and y represent the molar ratio of DMAPS to AA, and z represents the concentration of metal ions. (B) Photos of the transparency of D_1_A_2_Al_0.15_ and D_1_A_2_ at different temperatures. (C) The relationship between resistance and temperature of D_1_A_2_Al_0.15_ and D_1_A_2_ in the range of 0–50^o^C. (A–C) Adapted with permission from ref [29]. Copyright 2020 American Chemical Society. (D) The chemical structure of zwitterionic hydrogel (PMAA-co-DMAPS). The relationship between transparency and temperature of (E) UCST-type hydrogels and (F) LCST-type hydrogels with different monomer mass ratios (MAA/DMAPS). During heating at 25–55^o^C, the 2D synchronous and asynchronous spectrum of (G) UCST-type hydrogel (MAA/DMAPS = 2 : 5) and (H) LCST-type hydrogel (MAA/DMAPS = 5 : 2). Red is defined as positive intensity, and blue is defined as negative intensity. (D–H) Adapted with permission from ref [95]. Copyright 2018 American Chemical Society.

Temperature-sensitive hydrogel-based sensors find a wide range of applications in areas such as smart textiles, food packaging, and bio-medical engineering. The sensing mechanism of such sensors relies on the temperature-responsive characteristics of the hydrogel, which undergoes volume and property changes in response to temperature fluctuations. In order to overcome the limitations of traditional mercury and infrared thermometers, such as stiffness and safety concerns, it is favorable to prioritize the development of flexible, affordable, and bio-compatible wearable temperature sensors. A three-dimensional internal percolation network exists within the thermosensitive hydrogel, and its volume change can be reversed by water absorption or dehydration in response to temperature changes [95]. The hydrogel exhibits a phase transition at either the lower critical solution temperature (LCST) or the upper critical solution temperature (UCST), triggered by changes in its internal nanostructure or microstructure. This transition manifests as alterations in transparency, opacity, and conductivity. Hydrogels of the UCST and LCST types display contrasting sol-gel transformation behaviors. 3-Dimethyl (methacryloyloxyethyl) ammonium propanesulfonate (DMAPS), as a derivative of betaine is known for antimicrobial and nonfouling properties [95]. A P (DMAPS-co-AA)/Al^3+^ hydrogel with thermosensitivity was created by Tan *et al.* to illustrate the relationship between hydrogel transparency and temperature, and the UCST behavior of hydrogels was consequently related to the temperature-dependent ionic bonds generated between quaternary ammonium salts and sulfate or carboxylate salts (Fig. 10A) [29]. Temperature-dependent hydrogel transparency appeared to vary, which made it possible to visualize temperature sensors (Fig. 10B) [29]. The hydrogel's transition from transparent to opaque could be indirectly observed through the change in resistance due to temperature (Fig. 10C) [29]. Near UCST, the electrostatic interaction between polyanions and polycations weakens, causing significant changes in the optical transparency, conductivity, and stretchability of hydrogels. These unique properties allow for the development of digital visual wearable temperature sensors that can also monitor human stress and strain signals such as electrocardiogram [29].

Lei *et al*. prepared an amphoteric ionic hydrogel that could switch between UCST and LCST behaviors based on temperature changes [95]. Methacrylic acid (MAA) and DMAPS were copolymerized using a free radical polymerization process (Fig. 10D) [95]. Amphoteric ionic hydrogels with various MAA/DMAPS mass ratios displayed controllable critical transition behavior (Fig. 10E and F) [95]. A two-dimensional correlation spectroscopy study was conducted to comprehend the dynamic interaction mechanism behind various phase transition patterns (Fig. 10G and H) [95]. The findings revealed that the dynamic process of the UCST phase change was driven by the separation of SO_3_^−^ groups and the disruption of hydrogen bonds between the SO_3_^−^ group and water. Additionally, there was a decrease in the ion interaction between SO_3_^−^ and the quaternary ammonium group. The LCST phase behavior was driven by the breakage of hydrogen bonds and the loss of the C=O group from the ion phase, leading to the gradual dissociation of the SO_3_^−^ group. In addition to their response to thermal stimulation, UCST and LCST hydrogels also exhibit dynamic internal interactions that enable them to respond to mechanical stimulation. For instance, a sandwich-structured ionic skin based on zwitterionic hydrogel shows sensitive capacitive signals that accurately monitor the movements of robotic fingers [95].

The two most common responses of hydrogel temperature sensors are the resistance-temperature response [39] and capacitance-temperature response [96].


\begin{eqnarray*} TCR = \left[ {\left( {{R}_{\rm{t}} - {R}_0} \right)/{R}_0} \right]/\Delta T \end{eqnarray*}

The temperature resistance coefficient (*TCR*) is widely used to evaluate the sensitivity of temperature sensors, where *R*_t_ is the transient resistance, *R*_0_ is the primary resistance, and Δ*T* is the change in temperature. A silk protein-based hydrogel E-skin assembled with a Pt nanofibers network as a temperature sensor, for example, has a high temperature sensitivity (TCR = 0.205%°C^−1^) and a fast response time (<2 s) [97].

In the future, visual hydrogel temperature sensors will hold significant practical value due to their unique characteristics. These sensors utilize the local surface plasmon resonance, which involves the collective oscillation of conducting electrons in metal nanoparticles, resulting in visually appealing colors in plasma nanostructures. Additionally, temperature-sensitive fluorescent materials can be employed as temperature sensors to accurately detect organism temperatures. These sensors offer numerous advantages, including a high tissue penetration rate, minimal light-induced damage, exceptional precision, high sensitivity, and excellent stability.

## CONCLUSION AND PERSPECTIVE

In electronic skin applications, the mechanical and electrical properties of hydrogels are critical factors to consider. Developing a hydrogel sensor with superior sensing performance hinges on two key aspects: the toughening network and the conductive network. A thorough analysis was performed on the toughening and conductive networks of hydrogels, and an assessment was made on the structure, function, design methodology, and future potential of hydrogel sensors, considering the most recent progress in this domain. This summary of the available toughening methods and conductive networks can provide valuable guidance for designing and developing hydrogel sensors with diverse functionalities. There can be trade-offs between the mechanical strength and conductivity of hydrogels, and optimizing both properties simultaneously can be challenging.

First, hydrogels can be sensitive to environmental factors such as temperature, pH, and moisture content, which can impact their mechanical properties. It is important to carefully control these environmental factors when designing and using hydrogels in various applications, particularly those that require consistent and predictable mechanical properties. Developing new materials and techniques are required for creating hydrogels with high toughness, such as self-healing hydrogels or hydrogels with enhanced interpenetrating networks. In addition, exploring fabrication techniques, such as 3D printing or microfluidics, can lead to improved topology of hydrogels. These techniques can allow for precise control over the hydrogel structure and the incorporation of reinforcing agents or other materials to enhance its topology.

Second, to ensure accurate and immediate monitoring of external stimuli, it is crucial to consider the charge transfer, dispersion, and stability of the conductive network within the hydrogel matrix. The construction of conductive networks in conductive hydrogels often involves the use of ionic and electronic conductors. Both ionic and electron conductive hydrogels have their own advantages and disadvantages, and the choice between the two depends on the specific application. Ionic conductive hydrogels typically have lower conductivity than electron conductive hydrogels, but they are often more biocompatible and less toxic. Electron conductive hydrogels, on the other hand, can have higher conductivity but may be more difficult to fabricate and may have lower biocompatibility. Depending on the specific sensing requirements, researchers may opt for an appropriate toughening process and conductive network, undertaking extensive testing of conductive hydrogels to ensure their properties meet the requirements for specific applications, such as sensitivity and stability in biosensing.

An ideally high-quality hydrogel sensor must be resistant to the environment (especially hostile environments), has good moisture retention and anti-swelling properties, soft and human skin affinity, and exceptional mechanical and electrical performance. In application, there can be intelligent data processing to meet the needs of life, convenient and powerful data storage space, and a security guarantee. In addition, these sensors should be as inexpensive and pollution-free as possible. In the future, sensors will be systematized, innovative, micro-scale, integrated, intelligent, and industrialized. Hydrogel sensors still face other several challenges and obstacles.

First, owing to the unique properties of hydrogel materials, they are susceptible to environmental factors like humidity, temperature, light, ion strength, and pH. In hostile conditions, this can result in unstable performance, poor selectivity, low repeatability, and high costs. While addressing these issues, researchers often encounter a decline in the hydrogel's moisture retention, swelling, or integration performance. Different toughening or conducting methods can be explored to address the limitations of each method and enhance the overall properties of the hydrogel. For example, adjusting the ratios of cross-linking agents, water-absorbing agents, and moisturizing agents can help control the water absorption and swelling resistance of the hydrogel when high conductivity is not required.

Second, maintaining close contact between the hydrogel electrode and the skin can be challenging due to the skin's tendency to stretch, wrinkle, and bend with movement. Achieving a balance between adhesion and skin comfort is crucial to ensure that the hydrogel stays in place without obstructing sweat and air exchange. Making sensors thinner and more comfortable is another challenge. In order to meet the same requirements, further research is needed to ensure the sensitivity and accuracy of sensors. This involves developing new materials, designing and selecting suitable topology structures, and making surface modifications, among other things. Using biocompatible and biodegradable materials is a good option, but it can be less flexible. To improve flexibility, long-chain polymers can be a choice.

In addition to the challenges of achieving sensitivity, accuracy, and comfortability, there is also the issue of connecting hydrogels and wiring them to the integrated circuit that collects and processes signals at the back end. With the development of artificial intelligence, real-time wireless sensing has become an important trend in hydrogel-based sensor research. The integration of hydrogel sensors with flexible circuits and wireless communication modules is crucial to enable real-time monitoring and data transmission. Due to the softness and expandability of hydrogels, connecting and wiring becomes more complicated. Additionally, most integrated circuits are rigid, so developing flexible circuits that are compatible with hydrogel-based sensors is essential.

Solving these challenges will improve the performance of hydrogel-based sensors and expand their applications in various fields such as healthcare, robotics, and environmental monitoring. In the era of Internet of Things, information technology, and artificial intelligence, the use of wearable electronic skin made of hydrogel sensors is becoming increasingly important. However, in order to make these sensors a practical reality for everyday use, it is crucial to continue research and development toward effective integration, packaging, and other necessary technologies. Adding a wireless sensing module to the back end while maintaining user comfort requires further research and development. In addition, the packaging of hydrogel sensors in a biocompatible and durable material is essential to ensure their stability and reliability in various environmental conditions.

## Supplementary Material

nwad180_Supplemental_File

## References

[1] Song W , Gan B, Jiang T et al. Nanopillar arrayed triboelectric nanogenerator as a self-powered sensitive sensor for a sleep monitoring system. ACS Nano 2016; 10: 8097–103. 10.1021/acsnano.6b04344 27494273

[2] Liu J , Qu S, Suo Z et al. Functional hydrogel coatings. Natl Sci Rev 2021; 8: nwaa254. 10.1093/nsr/nwaa254 34691578 PMC8288423

[3] Kim J-H , Kim S-R, Kil H-J et al. Highly conformable, transparent electrodes for epidermal electronics. Nano Lett 2018; 18: 4531–40. 10.1021/acs.nanolett.8b01743 29923729

[4] Song W , Zhu J, Gan B et al. Flexible, stretchable, and transparent planar microsupercapacitors based on 3D porous laser-induced graphene. Small 2018; 14: 1702249. 10.1002/smll.201702249 29148212

[5] Zhang M , Yuan J. Graphene meta-aerogels: when sculpture aesthetic meets 1D/2D composite materials. Nano Res Energy 2022; 1: e9120035. 10.26599/NRE.2022.9120035

[6] Yu X-G , Li Y-Q, Zhu W-B et al. A wearable strain sensor based on a carbonized nano-sponge/silicone composite for human motion detection. Nanoscale 2017; 9: 6680–5. 10.1039/C7NR01011G 28485457

[7] Zhang D , Zhang Y, Lu W et al. Fluorescent hydrogel-coated paper/textile as flexible chemosensor for visual and wearable mercury(II) detection. Adv Mater Technol 2019; 4: 1800201. 10.1002/admt.201800201

[8] Xu C , Jiang D, Ge Y et al. A PEDOT: PSS conductive hydrogel incorporated with prussian blue nanoparticles for wearable and noninvasive monitoring of glucose. Chem Eng J 2022; 431: 134109. 10.1016/j.cej.2021.134109

[9] Zhou Q , Wu M, Zhang M et al. Graphene-based electrochemical capacitors with integrated high-performance. Mater Today Energy 2017; 6: 181–8. 10.1016/j.mtener.2017.09.015

[10] Chen J , An R, Han L et al. Tough hydrophobic association hydrogels with self-healing and reforming capabilities achieved by polymeric core-shell nanoparticles. Mater Sci Eng C 2019; 99: 460–7. 10.1016/j.msec.2019.02.005 30889720

[11] Dong X , Guo X, Liu Q et al. Strong and tough conductive organo-hydrogels via freeze-casting assisted solution substitution. Adv Funct Mater 2022; 32: 2203610. 10.1002/adfm.202203610

[12] Ye Y , Jiang F. Highly stretchable, durable, and transient conductive hydrogel for multi-functional sensor and signal transmission applications. Nano Energy 2022; 99: 107374. 10.1016/j.nanoen.2022.107374

[13] Jeon I , Cui J, Illeperuma WRK et al. Extremely stretchable and fast self-healing hydrogels. Adv Mater 2016; 28: 4678–83. 10.1002/adma.201600480 27061799

[14] Xu L , Huang Z, Deng Z et al. A transparent, highly stretchable, solvent-resistant, recyclable multifunctional ionogel with underwater self-healing and adhesion for reliable strain sensors. Adv Mater 2021; 33: 2105306. 10.1002/adma.202105306 34647370

[15] Yang G , Zhu K, Guo W et al. Adhesive and hydrophobic bilayer hydrogel enabled on-skin biosensors for high-fidelity classification of human emotion. Adv Funct Mater 2022; 32: 2200457. 10.1002/adfm.202200457

[16] Mredha MdTI , Guo YZ, Nonoyama T et al. A facile method to fabricate anisotropic hydrogels with perfectly aligned hierarchical fibrous structures. Adv Mater 2018; 30: 1704937. 10.1002/adma.201704937 29341264

[17] Wang F , Li Z, Guo J et al. Highly strong, tough, and stretchable conductive hydrogels based on silk sericin-mediated multiple physical interactions for flexible sensors. ACS Appl Polym Mater 2022; 4: 618–26. 10.1021/acsapm.1c01553

[18] Sun M , Bai R, Yang X et al. Hydrogel interferometry for ultrasensitive and highly selective chemical detection. Adv Mater 2018; 30: 1804916. 10.1002/adma.201804916 30252962

[19] Liu H , Wei S, Qiu H et al. Supramolecular hydrogel with orthogonally responsive R/G/B fluorophores enables multi-color switchable biomimetic soft skins. Adv Funct Mater 2022; 32: 2108830. 10.1002/adfm.202108830

[20] Li S , Yi J, Yu X et al. Preparation and characterization of acid resistant double cross-linked hydrogel for potential biomedical applications. ACS Biomater Sci Eng 2018; 4: 872–83. 10.1021/acsbiomaterials.7b00818 33418771

[21] An H , Bo Y, Chen D et al. Cellulose-based self-healing hydrogel through boronic ester bonds with excellent biocompatibility and conductivity. RSC Adv 2020; 10: 11300–10. 10.1039/C9RA10736C 35495323 PMC9050428

[22] Yin M-J , Zhang Y, Yin Z et al. Micropatterned elastic gold-nanowire/polyacrylamide composite hydrogels for wearable pressure sensors. Adv Mater Technol 2018; 3: 1800051. 10.1002/admt.201800051

[23] Chen G , Huang J, Gu J et al. Highly tough supramolecular double network hydrogel electrolytes for an artificial flexible and low-temperature tolerant sensor. J Mater Chem A 2020; 8: 6776–84. 10.1039/D0TA00002G

[24] Lu B , Yuk H, Lin S et al. Pure PEDOT: PSS hydrogels. Nat Commun 2019; 10: 1043. 10.1038/s41467-019-09003-5 30837483 PMC6401010

[25] Liu X , Wu J, Qiao K et al. Topoarchitected polymer networks expand the space of material properties. Nat Commun 2022; 13: 1622. 10.1038/s41467-022-29245-0 35338139 PMC8956700

[26] Gogurla N , Roy B, Min K et al. A skin-inspired, interactive, and flexible optoelectronic device with hydrated melanin nanoparticles in a protein hydrogel-elastomer hybrid. Adv Mater Technol 2020; 5: 1900936. 10.1002/admt.201900936

[27] Kim S-K , Lee G-H, Jeon C et al. Bimetallic nanocatalysts immobilized in nanoporous hydrogels for long-term robust continuous glucose monitoring of smart contact lens. Adv Mater 2022; 34: 2110536. 10.1002/adma.202110536 PMC10782562 35194844

[28] Tsai M-S , Shen T-L, Wu H-M et al. Self-powered, self-healed, and shape-adaptive ultraviolet photodetectors. ACS Appl Mater Interfaces 2020; 12: 9755–65. 10.1021/acsami.9b21446 32013376

[29] Tan Y , Zhang Y, Zhang Y et al. Dual cross-linked ion-based temperature-responsive conductive hydrogels with multiple sensors and steady electrocardiogram monitoring. Chem Mater 2020; 32: 7670–8. 10.1021/acs.chemmater.0c01589

[30] Tie J , Rong L, Liu H et al. An autonomously healable, highly stretchable and cyclically compressible, wearable hydrogel as a multimodal sensor. Polym Chem 2020; 11: 1327–36. 10.1039/C9PY01737B

[31] Pan Z , Kang X, Zeng Y et al. A mannosylated PEI-CPP hybrid for TRAIL gene targeting delivery for colorectal cancer therapy. Polym Chem 2017; 8: 5275–85. 10.1039/C7PY00882A

[32] Li Y , Yan J, Liu Y et al. Super tough and intelligent multibond network physical hydrogels facilitated by Ti_3_C_2_T*_x_*MXene nanosheets. ACS Nano 2022; 16: 1567–77. 10.1021/acsnano.1c10151 34958558

[33] Tang L , Wu S, Xu Y et al. High toughness fully physical cross-linked double network organohydrogels for strain sensors with anti-freezing and anti-fatigue properties. Mater Adv 2021; 2: 6655–64. 10.1039/D1MA00618E

[34] Huang G , Tang Z, Peng S et al. Modification of hydrophobic hydrogels into a strongly adhesive and tough hydrogel by electrostatic interaction. Macromolecules 2022; 55: 156–65. 10.1021/acs.macromol.1c01115

[35] Sun H , Zhao Y, Wang C et al. Ultra-stretchable, durable and conductive hydrogel with hybrid double network as high performance strain sensor and stretchable triboelectric nanogenerator. Nano Energy 2020; 76: 105035. 10.1016/j.nanoen.2020.105035

[36] Yu J , Xu K, Chen X et al. Highly stretchable, tough, resilient, and antifatigue hydrogels based on multiple hydrogen bonding interactions formed by phenylalanine derivatives. Biomacromolecules 2021; 22: 1297–304. 10.1021/acs.biomac.0c01788 33577294

[37] Li H , Zheng H, Tan YJ et al. Development of an ultrastretchable double-network hydrogel for flexible strain sensors. ACS Appl Mater Interfaces 2021; 13: 12814–23. 10.1021/acsami.0c19104 33427444

[38] Qi C , Dong Z, Huang Y et al. Tough, anti-swelling supramolecular hydrogels mediated by surfactant-polymer interactions for underwater sensors. ACS Appl Mater Interfaces 2022; 14: 30385–97. 10.1021/acsami.2c06395 35737578

[39] Wang Y , Chen F, Liu Z et al. A highly elastic and reversibly stretchable all-polymer supercapacitor. Angew Chem Int Ed 2019; 58: 15707–11. 10.1002/anie.201908985 31441591

[40] Li S , Gao Y, Jiang H et al. Tough, sticky and remoldable hydrophobic association hydrogel regulated by polysaccharide and sodium dodecyl sulfate as emulsifiers. Carbohydr Polym 2018; 201: 591–8. 10.1016/j.carbpol.2018.08.100 30241857

[41] Zheng H , Chen M, Sun Y et al. Self-healing, wet-adhesion silk fibroin conductive hydrogel as a wearable strain sensor for underwater applications. Chem Eng J 2022; 446: 136931. 10.1016/j.cej.2022.136931

[42] Xu G , Zhang J, Jia R et al. Topological effects of dendronized polymers on their thermoresponsiveness and microconfinement. Macromolecules 2022; 55: 630–42. 10.1021/acs.macromol.1c02066

[43] Tong QB , Du C, Wei Z et al. Synergic influences of network topologies and associative interactions on the microstructures and bulk performances of hydrogels. J Mater Chem B 2021; 9: 9863–73. 10.1039/D1TB02114A 34849519

[44] Kwon J , Suh YD, Lee J et al. Recent progress in silver nanowire based flexible/wearable optoelectronics. J Mater Chem C 2018; 6: 7445–61. 10.1039/C8TC01024B

[45] Jiang P-P , Qin H, Dai J et al. Ultrastretchable and self-healing conductors with double dynamic network for omni-healable capacitive strain sensors. Nano Lett 2022; 22: 1433–42. 10.1021/acs.nanolett.1c03618 34747171

[46] Chen J , An R, Han L et al. Tough hydrophobic association hydrogels with self-healing and reforming capabilities achieved by polymeric core-shell nanoparticles. Mater Sci Eng C 2019; 99: 460–7. 10.1016/j.msec.2019.02.005 30889720

[47] Yu W , Deschaume O, Dedroog L et al. Light-addressable nanocomposite hydrogels allow plasmonic actuation and *in situ* temperature monitoring in 3D cell matrices. Adv Funct Mater 2022; 32: 2108234. 10.1002/adfm.202108234

[48] Ni J , Lin S, Qin Z et al. Strong fatigue-resistant nanofibrous hydrogels inspired by lobster underbelly. Matter 2021; 4: 1919–34. 10.1016/j.matt.2021.03.023

[49] Wang J , Wu B, Wei P et al. Fatigue-free artificial ionic skin toughened by self-healable elastic nanomesh. Nat Commun 2022; 13: 4411. 10.1038/s41467-022-32140-3 35906238 PMC9338060

[50] Miao Y , Xu M, Zhang L. Electrochemistry-induced improvements of mechanical strength, self-healing, and interfacial adhesion of hydrogels. Adv Mater 2021; 33: 2102308. 10.1002/adma.202102308 34418178

[51] Enas MA . Hydrogel: preparation, characterization, and applications: a review. J Adv Res 2015; 6: 105–21. 25750745 10.1016/j.jare.2013.07.006 PMC4348459

[52] Chen C , Wang Y, Zhou T et al. Toward strong and tough wood-based hydrogels for sensors. Biomacromolecules 2021; 22: 5204–13. 10.1021/acs.biomac.1c01141 34787399

[53] Han L , Yan L, Wang M et al. Transparent, adhesive, and conductive hydrogel for soft bioelectronics based on light-transmitting polydopamine-doped polypyrrole nanofibrils. Chem Mater 2018; 30: 5561–72. 10.1021/acs.chemmater.8b01446

[54] Zhang S , Chen Y, Liu H et al. Room-temperature-formed PEDOT: PSS hydrogels enable injectable, soft, and healable organic bioelectronics. Adv Mater 2020; 32: 1904752. 10.1002/adma.201904752 PMC6946856 31657081

[55] Shen Z , Zhang Z, Zhang N et al. High-stretchability, ultralow-hysteresis conducting polymer hydrogel strain sensors for soft machines. Adv Mater 2022; 34: 2203650. 10.1002/adma.202203650 35726439

[56] Lee YY , Kang HY, Gwon SH et al. A strain-insensitive stretchable electronic conductor: PEDOT: PSS/acrylamide organogels. Adv Mater 2016; 28: 1636–43. 10.1002/adma.201504606 26684678

[57] Ge G , Lu Y, Qu X et al. Muscle-inspired self-healing hydrogels for strain and temperature sensor. ACS Nano 2020; 14: 218–28. 10.1021/acsnano.9b07874 31808670

[58] Zhu J , Huang X, Song W. Physical and chemical sensors on the basis of laser-induced graphene: mechanisms, applications, and perspectives. ACS Nano 2021; 15: 18708–41. 10.1021/acsnano.1c05806 34881870

[59] Zhang Y , Liang B, Jiang QF et al. Flexible and wearable sensor based on graphene nanocomposite hydrogels. Smart Mater Struct 2020; 29:075027. 10.1088/1361-665X/ab89ff

[60] Wang Z , Hao Z, Yang C et al. Ultra-sensitive and rapid screening of acute myocardial infarction using 3D-affinity graphene biosensor. Cell Rep Phys Sci 2022; 3: 100855. 10.1016/j.xcrp.2022.100855

[61] Stankovich S , Dikin DA, Dommett GHB et al. Graphene-based composite materials. Nature 2006; 442: 282–6. 10.1038/nature04969 16855586

[62] Yue L , Zhang X, Li W et al. Quickly self-healing hydrogel at room temperature with high conductivity synthesized through simple free radical polymerization. J Appl Polym Sci 2019; 136: 47379. 10.1002/app.47379

[63] Lin M , Zheng Z, Yang L et al. A high-performance, sensitive, wearable multifunctional sensor based on rubber/CNT for human motion and skin temperature detection. Adv Mater 2022; 34: 2107309. 10.1002/adma.202107309 34648668

[64] Tai Y , Mulle M, Ventura IA et al. A highly sensitive, low-cost, wearable pressure sensor based on conductive hydrogel spheres. Nanoscale 2015; 7: 14766–73. 10.1039/C5NR03155A 26288336

[65] Wei Z , Wang J, Guo S et al. Towards highly salt-rejecting solar interfacial evaporation: photothermal materials selection, structural designs, and energy management. Nano Res Energy 2022; 1: e9120014. 10.26599/NRE.2022.9120014

[66] Di X , Ma Q, Xu Y et al. High-performance ionic conductive poly(vinylalcohol) hydrogels for flexible strain sensors based on a universal soaking strategy. Mater Chem Front 2021; 5: 315–23. 10.1039/D0QM00625D

[67] Dai S , Wang S, Yan H et al. Stretchable and self-healable hydrogel-based capacitance pressure and strain sensor for electronic skin systems. Mater Res Express 2019; 6: 0850b9. 10.1088/2053-1591/ab2320

[68] Liu C , Zhang HJ, You X et al. Electrically conductive tough gelatin hydrogel. Adv Electron Mater 2020; 6: 2000040. 10.1002/aelm.202000040

[69] Ding Y , Zhang J, Chang L et al. Preparation of high-performance ionogels with excellent transparency, good mechanical strength, and high conductivity. Adv Mater 2017; 29: 1704253. 10.1002/adma.201704253 29083496

[70] Villa SM , Mazzola VM, Santaniello T et al. Soft piezoionic/piezoelectric nanocomposites based on ionogel/BaTiO_3_ nanoparticles for low frequency and directional discriminative pressure sensing. ACS Macro Lett 2019; 8: 414–20. 10.1021/acsmacrolett.8b01011 35651125

[71] Huang H , Han L, Li J et al. Super-stretchable, elastic and recoverable ionic conductive hydrogel for wireless wearable, stretchable sensor. J Mater Chem A 2020; 8: 10291–300. 10.1039/D0TA02902E

[72] Peng X , Liu H, Yin Q et al. A zwitterionic gel electrolyte for efficient solid-state supercapacitors. Nat Commun 2016; 7: 11782. 10.1038/ncomms11782 27225484 PMC4894970

[73] Ren J , Liu Y, Wang Z et al. An anti-swellable hydrogel strain sensor for underwater motion detection. Adv Funct Mater 2022; 32: 2107404. 10.1002/adfm.202107404

[74] Shen K , Xu K, Zhang M et al. Multiple hydrogen bonds reinforced conductive hydrogels with robust elasticity and ultra-durability as multifunctional ionic skins. Chem Eng J 2023; 451: 138525. 10.1016/j.cej.2022.138525

[75] Rogers JA , Someya T, Huang Y. Materials and mechanics for stretchable electronics. Science 2010; 327: 1603–7. 10.1126/science.1182383 20339064

[76] Ma M , Shang Y, Shen H et al. Highly transparent conductive ionohydrogel for all-climate wireless human-motion sensor. Chem Eng J 2021; 420: 129865. 10.1016/j.cej.2021.129865

[77] Lin G , Si M, Wang L et al. Dual-channel flexible strain sensors based on mechanofluorescent and conductive hydrogel laminates. Adv Opt Mater 2022; 10: 2102306. 10.1002/adom.202102306

[78] Kwon JH , Kim YM, Moon HC. Porous ion gel: a versatile ionotronic sensory platform for high-performance, wearable ionoskins with electrical and optical dual output. ACS Nano 2021; 15: 15132–41. 10.1021/acsnano.1c05570 34427425

[79] Wei Y , Xiang L, Zhu P et al. Multifunctional organohydrogel-based ionic skin for capacitance and temperature sensing toward intelligent skin-like devices. Chem Mater 2021; 33: 8623–34. 10.1021/acs.chemmater.1c01904

[80] Kim DW , Song K-IL, Seong D et al. Electrostatic-mechanical synergistic in situ multiscale tissue adhesion for sustainable residue-free bioelectronics interfaces. Adv Mater 2022; 34: 2105338. 10.1002/adma.202105338 34783075

[81] Villa SM , Mazzola VM, Santaniello T et al. Soft piezoionic/piezoelectric nanocomposites based on ionogel/BaTiO_3_ nanoparticles for low frequency and directional discriminative pressure sensing. ACS Macro Lett 2019; 8: 414–20. 10.1021/acsmacrolett.8b01011 35651125

[82] Sun D , Feng Y, Sun S et al. Transparent, self-adhesive, conductive organohydrogels with fast gelation from lignin-based self-catalytic system for extreme environment-resistant triboelectric nanogenerators. Adv Funct Mater 2022; 32: 2201335. 10.1002/adfm.202201335

[83] Zhang J , Zeng L, Qiao Z et al. Functionalizing double-network hydrogels for applications in remote actuation and in low-temperature strain sensing. ACS Appl Mater Interfaces 2020; 12: 30247–58. 10.1021/acsami.0c10430 32525651

[84] Das R , Bej S, Murmu NC et al. Selective recognition of ammonia and aliphatic amines by C-N fused phenazine derivative: a hydrogel based smartphone assisted ‘optoelectronic nose’ for food spoilage evaluation with potent anticounterfeiting activity and a potential prostate cancer biomarker sensor. Anal Chim Acta 2022; 1202: 339597. 10.1016/j.aca.2022.339597 35341532

[85] Bej S , Mandal S, Mondal A et al. Solvothermal synthesis of high-performance d^10^-mofs with hydrogel membranes @ ‘turn-on’ monitoring of formaldehyde in solution and vapor phase. ACS Appl Mater Interfaces 2021; 13: 25153–63. 10.1021/acsami.1c05998 34011156

[86] Xu J-Q , Liu Y-L, Wang Q et al. Photocatalytically renewable micro-electrochemical sensor for real-time monitoring of cells. Angew Chem Int Ed 2015; 54: 14402–6. 10.1002/anie.201507354 26768108

[87] Das R , Bej S, Ghosh D et al. Stimuli-responsive discriminative detection of Cu^2+^ and Hg^2+^ with concurrent sensing of S^2−^ from aqueous medium and bio-fluids by C-N fused azophenine functionalized ‘smart’ hydrogel assay @ A potential biomarker sensor for Wilson's disease. Sens Actuators B Chem 2021; 341: 129925. 10.1016/j.snb.2021.129925

[88] Kim S-K , Lee G-H, Jeon C et al. Bimetallic nanocatalysts immobilized in nanoporous hydrogels for long-term robust continuous glucose monitoring of smart contact lens. Adv Mater 2022; 34: 2110536. 10.1002/adma.202110536 PMC10782562 35194844

[89] Wang Y , Yu Y, Guo J et al. Bio-inspired stretchable, adhesive, and conductive structural color film for visually flexible electronics. Adv Funct Mater 2020; 30: 2000151. 10.1002/adfm.202000151

[90] Weng G , Thanneeru S, He J. Dynamic coordination of Eu-iminodiacetate to control fluorochromic response of polymer hydrogels to multistimuli. Adv Mater 2018; 30: 1706526. 10.1002/adma.201706526 29334152

[91] Zhang Z , Chen Z, Wang Y et al. Bioinspired conductive cellulose liquid-crystal hydrogels as multifunctional electrical skins. Proc Natl Acad Sci USA 2020; 117: 18310–6. 10.1073/pnas.2007032117 32675247 PMC7414159

[92] Hazra A , Mondal U, Mandal S et al. Advancement in functionalized luminescent frameworks and their prospective applications as inkjet-printed sensors and anti-counterfeit materials. Dalton Trans 2021; 50: 8657–70. 10.1039/D1DT00705J 34060577

[93] Yan W , Ma C, Cai X et al. Self-powered and wireless physiological monitoring system with integrated power supply and sensors. Nano Energy 2023; **108**: 108203. 10.1016/j.nanoen.2023.108203

[94] Ding B , Zeng P, Huang Z et al. A 2D material-based transparent hydrogel with engineerable interference colours. Nat Commun 2022; 13: 1212. 10.1038/s41467-021-26587-z 35260559 PMC8904793

[95] Lei Z , Wu P. Zwitterionic skins with a wide scope of customizable functionalities. ACS Nano 2018; 12: 12860–8. 10.1021/acsnano.8b08062 30444602

[96] Xue B , Sheng H, Li Y et al. stretchable and self-healable hydrogel artificial skin. Natl Sci Rev 2022; 9: nwab147. 10.1093/nsr/nwab147 35974839 PMC9375542

[97] Huang J , Xu Z, Qiu W et al. Stretchable and heat-resistant protein-based electronic skin for human thermoregulation. Adv Funct Mater 2020; 30: 1910547. 10.1002/adfm.201910547

