# Supplementary material for: Pathways toward wearable and high-performance sensors based on hydrogels: toughening networks and conductive networks
Source: Natl Sci Rev. 2023 Jun 22;10(9):nwad180. doi: 10.1093/nsr/nwad180 (PMC10411675; doi:10.1093/nsr/nwad180)
Supplement: nwad180_Supplemental_File [file nwad180_supplemental_file.pdf]

Supporting Information

Pathways towards wearable and high-performance sensors based on hydrogels: toughening networks and conductive networks

Junbo Zhu[1,†], Jingchen Tao[1,†], Wei Yan[1] and Weixing Song[1,*]

[1]Beijing Key Laboratory for Optical Materials and Photonic Devices, Department of Chemistry, Capital Normal University, Beijing 100048, China

***Corresponding author**. E-mail: songwx@cnu.edu.cn

[†]Equally contributed to this work.

Contents

1. The Development of Hydrogel Sensors..2
2. The Properties for Hydrogel Sensors...3
3. References..9

1. The Development of Hydrogel Sensors

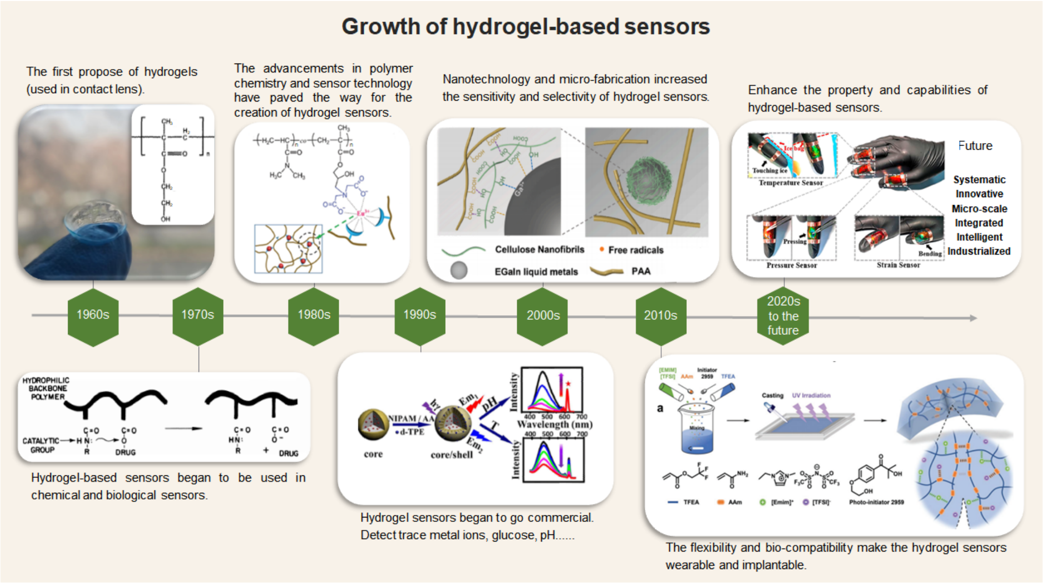

Figure S1. The growth of hydrogel sensors [1-14]. Adapted with permissions from refs. [1-6].

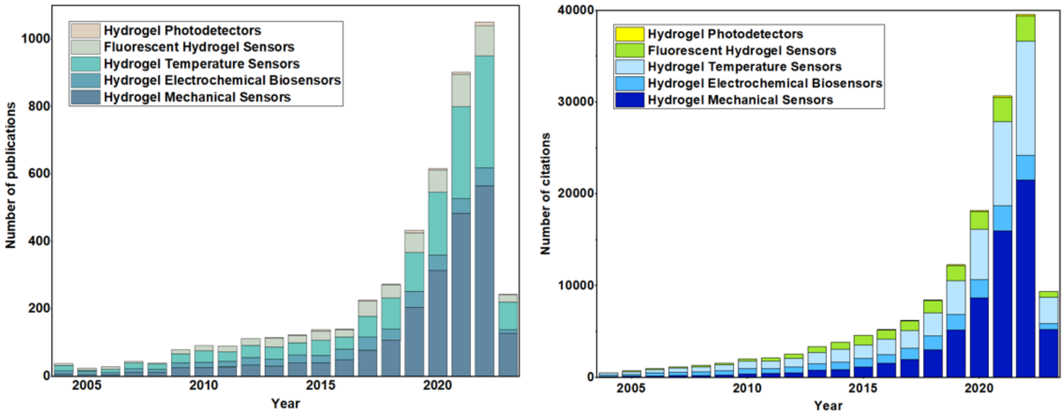

Figure S2. Total number of publications and citation papers related to hydrogel-based sensors in the last two decades according to the Web of Science, searched on April 1, 2023.

2. The Properties for Hydrogel Sensors

Table 1. This table summarizes the various strategies, including the use of substrates, additives, intensity, elongation at break, and methods. The 'intensity' and 'elongation at break' listed in the table represent the highest values reported in the literature and do not have a correlating relationship.

Strategy	Substrate[a]	Additive[a]	Intensity (σb) (MPa)	elongation at break (Ɛb) (%)	Method	Refs.
Double-network	PAM	PEDOT: PSS/ Gelatin	0.2	2200	The hydrogen bonds between PAM and PAM, PSS and gelatin networks are sacrificed.	[15]
		GF/CA/ Chitosan	0.21	600.36	PAM network remains intact and has a stable defect.	[16]
	PNN	TPP/GA/ Chitosan	0.072	1200	Covalent cross-linking as a second network.	[17]
	PAA	SA/AM	0.21	4200	The dynamic interaction keeps the polymer chain stable.	[18]
Ion coordination	PVA	SS/Na_3Cit	4.42	>500	Hydrogen bond and ion coordination.	[19]
		AM/Fe^{3+}	0.37	700	The combined effect of PAM chain network and chelation.	[20]
	PAA	Agar/Fe^{3+}	0.0696	3174.3	Hydrogen bond and ion coordination.	[21]
		MXene/Fe^{3+}	10.4	3080	Hydrogen bond and ion coordination.	[22]
	PAM	PBA-IL/ CNFs	0.349	1810	CNFs and PAM/PBA-IL serve as sacrificial keys.	[23]
		APBA/NaCl	0.21	1600	NaCl increases hydrogen bond fracture strain.	[24]
Hydrophobic association	PAA	[BMIM]TFSI	0.1156	1145.7	The reversible hydrophobic interaction between the hydrophobic networks eliminates energy.	[25]
		LMA/CTAB	1.6	>900	The synergistic effect of electrostatic interaction and hydrophobic association of Copolymers.	[26]
	PAM	PSBMA/ Al^{3+}/ Laponite XLG	0.8	690	Hydrogen bond and electrostatic bond form seamless adhesion, hydrophobicity restrain adhesion decay.	[27]
	PBA	DMAEMA/ MMA	0.1034	1560	The physical crosslink point acts as a sacrificial bond.	[28]
Topology	PAA	PNIPAM (GN)	4	470	Different network topologies result in different density and strength of hydrogen bonds, different microstructure and macroscopic properties	[29]
		PNIPAM (SN)	0.073	60		
		PNIPAM (IPN)	0.37	474		
	PAM	PAMPS	0.04	≈350	Topological entanglement.	[30]
	PAMPS	IL/PDMAA	0.28	800	Elastic covalent cross-linked interpenetrating microsphere network as a sacrificial structure.	[31]

Introduction of nanostructures	PAA	LMNPs/ CNFs	0.2	2950	CFS stabilized LMNPs, hydrogen bond interaction and pure physical crosslinking	[4]
	PVA	CNT/AgNW	-	1000	SWCNT promotes the self-healing of hydrogel and improves its toughness.	[32]
	PAM	AgNW/rGO	3.0	3250	π-π interaction, Ag-SR coordination and mechanical friction.	[33]
		PSNPs	0.3	1600	Hydrogen bonding and double physical cross-linking of hydrophobic chains	[34]
Functionalized modification	PAM	κ-carrageenan, Fe^{3+}	0.32	4600	electrochemistry functionalization strategy enhances the adhesion interaction between hydrogel and smooth substrate.	[35]
		CNF	13.43	15.89	The UV grafting effect caused favorable interfacial interactions between wood and PAM, enhanced tensile properties of the hydrogel.	[36]
Engineering Methods	PVA	HA	23.5	2900	Using a freezing-assisted salting-out treatment.	[37]
			8.4	-	Electrospinning, welding and dry annealing.	[38]
		FC-EtFe	6.5	1710	The anisotropic structure is combined with the reinforcement and toughening.	[39]

[a]Note: poly (N-isopropylacrylamide-co-N,N'-diethylacrylamide) (PNN), phenylboronic acid (PBA), poly (2-acrylamido-2-methyl-1-propanesulfonic acid) (PAMPS), graphene foam (GF), calcium-alginate (CA), tripolyphosphate (TPP), glutaralde-hyde (GA), sodium alginate (SA), 3-acrylamidophenylboronic acid (APBA), 1-butyl-3-methylimidazolium bis(trifluoromethanesulfonyl) imide ([BMIM]TFSI), laurylmethacrylate (LMA), cetyltrimethylammoniumbromide (CTAB), poly [2-(methacryloyloxy)ethyl] dimethyl-(3-sulfopropyl) ammonium hydroxide (PSBMA), 2-(dimethylamino) ethyl methacrylate (DMAEMA), methacrylic acid (MMA), poly (N-isopropylacrylamide) (PNIPAM), poly (N,N-dimethylacrylamide) (PDMAA), polystyrene (PS), Hyaluronic acid (HA), the highly cross-linked PVA organo-hydrogel by the FASS strategy (FC-EtFe).

Table 2. The comparison of the electrical conductivity of various hydrogel substrates created by mixing them with electronic conductors and ionic conductors.

Strategy	Substrate[a]	Additive[a]	Conductivity ($S\ m^{-1}$)	Method	Refs.
Electronic conductors	PAM	CNFs	5×10^{-2}	Naturally arranged nanochannels and negatively charged surfaces can transport ions.	[40]
	PNIPAM	CNT, clay	0.20	3D-printed grid scaffolds facilitate electron transfer.	[41]
	PAA	rGO-AM	27.2	The reduced graphene oxide sheet interacts with the hydrophilic group to provide free electrons.	[42]
	PS	SWNT	0.10	Graphene fillers are electrically conductive.	[43]
	PDMS	AgNW, MNP	3.76×10^{-4}	MNPs are similar to *n*-type semiconductors. Illumination increases carrier generation and electrical conductivity.	[44]
	PPy	PDA, PAM	12	Nanofibers provide a more complete conductive path.	[45]
		TOCNFs, PAA	3.90	The highly water-retaining structure promotes ion and electron transfer.	[46]
	PEDOT：PSS	-	10	PEDOT: PSS provides a conductive network.	[47]
		PAA	1	EG rarely evaporates in ambient air to ensure electrical stability.	[48]
		DMSO	20	PEDOT: PSS mixed with DMSO gives electrical properties.	[49]
	PEDOT: guar gum	[BMIM]Cl	1	It combines the redox properties of Conductive polymer with the ionic conductivity of ionic liquids	[50]
	PSS	UPy	13	Electron conduction AIDS ion transport.	[51]
Ionic conductors	PAA	LMNPs，CNFs	0.45	As the amounts of nanoparticles increases, the quantum tunneling effect becomes stronger, and so the conductivity improves.	[4]
		[EMIM]Cl	3.89	Ionic liquids have charge transfer.	[52]
	PAMPS	[EMIM][DCA]	2.40	[EMIM][DCA] is physically located in a PAMPS network and is free to transport at applied voltage.	[53]
	P (DMAPS-co-AA)	PEGDA， Al^{3+}	1.34	Metal ions regulate electrical conductivity.	[54]
	PDMS	P (DMAPS-co-AA), [EMIM][OAc]	1.24	Ionic liquids have charge transfer.	[55]
	PVA	FC-EtFe	6.50	Fass strategy.	[39]
		HPC	3.40	More pores provide more space for ion migration.	[56]
	AS	Gelatin	3.40	The Ion-rich region caused by salt effect increases the conductivity.	[57]

[a]Note: poly [3-dimethyl (methacryloyloxyethyl) ammonium propane sulfonate-co-acrylic acid] (P (DMAPS-co-AA)), ammonium sulfate (AS), melanin nanoparticle (MNP), TEMPO-oxidized cellulose nanofibers (TOCNFs), 2-ureido-4[1H]-pyrimidinone (UPy), 1-ethyl-3-methylimidazolium dicyanamide ([EMIM][DCA]), poly (ethylene glycol) diacrylate (PEGDA).

Table 3. Selection of hydrogel substrates and additives for the development of various types of wearable sensors, with their properties and applications.

Type	Substrate[a]	Additive[a]	Property	Application of hydrogel	Refs.
Pressure sensor	PAM	PAA, Ca^{2+}	The sensitivity reaches 3.71 kPa^{-1} in the pressure range of 0-2.8 kPa.<br>The sensitivity reaches 0.69 kPa^{-1} in the pressure range of >14 kPa.	The composite hydrogel forms a micropore on the electrode, which can be used to fabricate a printable piezoresistive pressure sensor rapidly.	[11]
	PAA	Pe/Fe^{3+}	The sensitivity reaches 0.65 kPa^{-1} in the pressure range of <15 kPa.<br>The sensitivity reaches 0.24 kPa^{-1} in the pressure range of 15-25 kPa.	The hydrogel's stability, repeatability, and self-healing capabilities help realize capacitive pressure and strain sensors.	[58]
Strain sensors	PVA	SBMA, HEMA	GF = 0.82 in the range of 0-150% strain<br>GF = 1.323 in the range of 150-250% strain	The antiswelling property of the amphoteric ionic polymer is achieved by the elimination of water molecules caused by protonation.	[59]
		SS/Na_3Cit	GF = 0.8 in the range of 0-50% strain<br>GF = 1.4 when strain increased to300%	SS mediated multiple physical interactions and the coordination effect of Na^+ and Cit^{3-} ions.	[20]
	PAM	[EMIM]Cl	GF = 1.66 in the range of 0-200% strain<br>GF = 3.64 in the range of 200-600% strain<br>GF = 6.78 in the range of 600-800% strain	The strain sensor made of ionic hydrogel has the advantages of recoverability and anti-fatigue, superior sensitivity and durability.	[52]
	PAA	Agar, Fe^{3+}	GF = 0.06 in the range of 60-200% strain<br>GF = 0.348 in the range of 200-700% strain<br>GF = 0.83 in the range of 700-1000% strain	DN hydrogels incorporate the properties of high tensile strength and strain sensitivity.	[21]
Electrochemical bio-sensor	PVA	Chitosan, HA-Au@Pt BiNCs, GOx	The sensitivity was 27.8 μA mM^{-1} cm^{-2} at 1 mg dl^{-1} unit detection limit<br>The sensitivity was 65.3 μA mM^{-1} cm^{-2} at 0.1 mg dl^{-1} unit detection limit	The nanocatalysts facilitate rapid decomposi tion of hydrogen peroxide and nanoparticle-mediated charge transfer with drastically	[60]
	PEDOT: PSS	PBNPs, DMSO, HEMA	The sensitivity was 340.1 μA mM^{-1} cm^{-2} at 1-243 μM glucose concentration	PEDOT: PSS combines with Prussian blue nanoparticles (PBNPs) for noninvasive and continuous monitoring of glucose in the body.	[14]

			The sensitivity was 184.3 μA mM^{-1} cm^{-2} at 243-3243 μM glucose concentration		
	PACA	HPC, CNTs	HPC could still form photonic liquid-crystal structure and show bright structural color, while the CNTs could enhance the saturation of the structural color and the PACA could locate the HPC assembled structure after polymerization.	Inspired by chameleons	[6]
	PNIPAM	Tb^{3+}, K_6APA, PNAGA	When the pH ranges from 5 to 9, the color changes from red to blue. When the hydration of EU^{3+}-K6APA complex decreased, the red fluorescence was enhanced. Tb^{3+} reacts with red fluorescent EU^{3+}-K6APA complex to form green complex.	In a reasonable material design, different types of fluorophore are organized into different polymer chains.	[61]
Fluorescent color-changing hydrogel sensor	PEGDA	CTO	When the LCST (≈32 °C) of PNIPAM is heated above, the red-violet fluorescence color is enhanced. From 20 ℃ to 76 ℃, PINK turns light green. As it warps, the color changes from green (0%) to purple (10%), then to third-order green (20%) and finally yellow (45%).	As an optical element, anisotropic hydrogel has the ability of light modulation.	[62]
	P (NDBCB-co-HEMA)	Hg^{2+}	Capillary force driving diffusion of Hg^{2+} solutions into hydrogel-coated film which results in desulfurization of the thiourea groups and subsequent cyclization to produce the corresponding blue.	The highly green-light-emitting polymer hydrogel coated paper chemosensor gradually becomes blue, generating Hg^{2+}-concentration-dependent fluorescence intensity and color change.	[7]
	PDA	PU	Owing to the flexibility and inverse opal structure of PU, the film exhibited stable stretchability and brilliant structural color.	Inspired by the adhesion features of mussels and the color shift mechanism of chameleons,	[63]
Hydrogel photodetector	PEGDA	CTO	Sensitive magnetic response, large and uniform optical anisotropy as well as the resultant multiple transmitted interference colors.	Peak wave length for each color is tuned via magnetically controlled alignment of 2D CTO materials during hydrogelation.	[62]
	PVA	Agarose	The photodetector enables 90% of	The self-powering capability greatly enhances the	[64]

			initial efficiency to be restored after five healing cycles, and each rapid healing time is suppressed to only 10 s.	sustainability of the device and the adaptability to wearable devices.	
	MC	MSNs, IR820	The release rate of DOX in PBS was 69.11% within 24 hours after irradiation. Under the irradiation of 808 nm laser, the GEL/IR_{820} showed a strong red signal in the irradiated region, while the non-irradiated region showed a green signal. The efficiency of killing tumor cells was nearly 100% within 5 min.	The introduction of a hydrogel network with NIR-reactive drug-release properties makes it possible for large doses of drugs to kill tumors.	[65]
Temperature sensors	PAM	CNF, TA, NaCl	It has a thermal response of 0.0149 C^{-1} in the temperature range of 25 ~ 90 ℃.	Ionic organic hydrogels can be used as skin-like thermistors to achieve early warning of high temperatures.	[66]
	PEGDA	P (DMAPS-co-AA), Al^{3+}	The temperature dependence of electrostatic interaction between cationic groups and anionic groups caused the hydrogels to have a very stable, reproducible, and visual temperature response.	Synchronously integrate physically and chemically dual cross linked, stable sensing, and multiple stimuli responses.	[54]
	XSBR	SSCNT	It has a thermal response of 0.01636 C^{-1} in the temperature range of 30 ~ 100 ℃.	Under the condition of alternating heat and cold, the relative change of resistance shows obvious difference.	[67]

[a]Note: poly (Acrylamide-co-Acrylic acid) (PACA), poly (1,8-naphthalimide-based monomer-co-2-hydroxyethyl methacrylate) (P (NDBCB-co-HEMA)), methylcellulose (MC), carboxylic styrene butadiene rubber (XSBR), methylated galacturonic acid (Pe), 2-(methacryloyloxy) ethyl]dimethyl-(3-sulfopropyl) ammonium hydroxide (SBMA), 2-hydroxyethyl methacrylate (HEMA), bimetallic nanocatalysts (BiNCs), prussian blue nanoparticles (PBNPs), hydroxypropyl cellulose (HPC), potassium 6-acrylamidopicolinate (K_6APA), poly (N-acryloyl glycinamide) (PNAGA), cobalt-doped titanium oxide (CTO), polyurethane (PU), mesoporous silica nanoparticles (MSNs), tannic acid (TA).

3. REFERENCES

1. Ratner BD and Hoffman AS. Synthetic hydrogels for biomedical applications. *ACS Symp Ser* 1976; **1:** 1-36.
2. Zhao Y, Shi C and Yang XD *et al.* pH- and temperature-sensitive hydrogel nanoparticles with dual photoluminescence for bioprobes. *ACS nano* 2016 **10:** 5856-63.
3. Weng G, Thanneeru S and He J. Dynamic coordination of Eu-iminodiacetate to control fluorochromic response of polymer hydrogels to multistimuli. *Adv Mater* 2018; **30:** 1706526.
4. Ye Y and Jiang F. Highly stretchable, durable, and transient conductive hydrogel for multi-functional sensor and signal transmission applications. *Nano Energy* 2022; **99:** 107374.
5. Xu LG, Huang ZK and Deng ZS *et al.* A transparent, highly stretchable, solvent-resistant, recyclable multifunctional ionogel with underwater self‐healing and adhesion for reliable strain sensors. *Adv Mater* 2021 **33:** 2105306.
6. Zhang Z, Chen Z and Wang Y *et al.* Bioinspired conductive cellulose liquid-crystal hydrogels as multifunctional electrical skins. *Proc Natl Acad Sci U S A* 2018; **117:** 18310-6.
7. Wichterle O and Lim D. Hydrophilic gels for biological use. *Nature* 1960; **185:** 117-8.
8. van den Vlekkert H, Francis C and Grisel A *et al.* Solvent polymeric membranes combined with chemical solid-state sensors. *Analyst* 1988; **113:** 1029-33.
9. Holtz J H and Asher S A. Polymerized colloidal crystal hydrogel films as intelligent chemical sensing materials. *Nature* 1997; **389:** 829-32.
10. Zhang X, Guo Q and Cui D. Recent advances in nanotechnology applied to biosensors. *Sensors* 2009; **9:** 1033-53.
11. Chen G, Huang J and Gu J *et al.* Highly tough supramolecular double network hydrogel electrolytes for an artificial flexible and low-temperature tolerant sensor. *J Mater Chem A* 2020; **8:** 6776-84.
12. Zhang D, Zhang Y and Lu W *et al.* Fluorescent hydrogel-coated paper/textile as flexible chemosensor for visual and wearable mercury(II) detection. *Adv Mater Technol* 2019; **4:** 1800201.
13. Zhang M and Yuan JY. Graphene meta-aerogels: When sculpture aesthetic meets 1D/2D composite materials. *Nano Res Energy* 2022; **1:** e9120035.
14. Xu C, Jiang D and Ge Y *et al.* A PEDOT:PSS conductive hydrogel incorporated with Prussian blue nanoparticles for wearable and noninvasive monitoring of glucose. *Chem Eng J* 2022; **431:** 134109.
15. Sun H, Zhao Y and Wang C *et al.* Ultra-Stretchable, durable and conductive hydrogel with hybrid double network as high performance strain sensor and stretchable triboelectric nanogenerator. *Nano Energy* 2020; **76:** 105035.
16. Cai Y, Qin J and Li W *et al.* A stretchable, conformable, and biocompatible graphene strain sensor based on a structured hydrogel for clinical application. *J Mater Chem A* 2019; **7:** 27099-109.
17. Dinh Xuan H, Timothy B and Park H *et al.* Super stretchable and durable electroluminescent devices based on double‐network ionogels. *Adv Mater* 2021; **33:** 2008849.
18. Huang and Hailong *et al.* Super-stretchable, elastic and recoverable ionic conductive hydrogel for wireless wearable, stretchable sensor. *J Mater Chem A* 2020; **8:** 10291-300.
19. Wang FF, Li Z and Guo JQ *et al.* Highly strong, tough, and stretchable conductive hydrogels based on silk sericin-mediated multiple physical interactions for flexible sensors. *ACS Appl Polym Mater* 2022; **4:** 618-26.

20. Pan Z, Kang X and Zeng Y *et al.* A mannosylated PEI-CPP hybrid for TRAIL gene targeting delivery for colorectal cancer therapy. *Polym Chem* 2017; **8:** 5275-85.
21. Li H, Zheng H and Tan YJ *et al.* Development of an ultrastretchable double-network hydrogel for flexible strain sensors. *ACS Appl Mater Interfaces* 2021; **13:** 12814-23.
22. Li Y, Yan J and Liu Y *et al.* Super tough and intelligent multibond network physical hydrogels facilitated by $Ti_3C_2T_x$ MXene nanosheets. *ACS Nano* 2022; **16:** 1567-77.
23. Yao X, Zhang S and Qian L *et al.* Super stretchable, self‐healing, adhesive ionic conductive hydrogels based on tailor‐made ionic liquid for high‐performance strain sensors. *Adv Funct Mater* 2022; **32:** 2204565.
24. Xu K, Shen KX and Yu J *et al.* Ultradurable noncovalent cross-linked hydrogels with low hysteresis and robust elasticity for flexible electronics. *Chem Mater* 2022; **34:** 3311-22.
25. Wei J, Zheng Y and Chen T. A fully hydrophobic ionogel enables highly efficient wearable underwater sensors and communicators *Mater Horiz* 2021; **8:** 2761-70.
26. Qi C, Dong Z and Huang Y *et al.* Tough, anti-swelling supramolecular hydrogels mediated by surfactant-polymer interactions for underwater sensors. *ACS Appl Mater Interfaces* 2022; **14:** 30385-97.
27. Yang G, Zhu K and Guo W *et al.* Adhesive and hydrophobic bilayer hydrogel enabled on‐skin biosensors for high‐fidelity classification of human emotion. *Adv Funct Mater* 2022; **32:** 2200457.
28. Chen H, Hao B and Ge P *et al.* Highly stretchable, self-healing, and 3D printing prefabricatable hydrophobic association hydrogels with the assistance of electrostatic interaction. *Polym Chem* 2020; **11:** 4741-8.
29. Tong Q B, Du C and Wei Z *et al.* Synergic influences of network topologies and associative interactions on the microstructures and bulk performances of hydrogels. *J Mater Chem B* 2021; **9:** 9863-73.
30. Liu X, Wu J and Qiao K *et al.* Topoarchitected polymer networks expand the space of material properties. *Nat Commun* 2022; **13:** 1622.
31. Li W, Li L and Zheng S *et al.* Recyclable, healable, and tough ionogels insensitive to crack propagation. *Adv Mater* 2022; **34:** 2203049.
32. Cai G, Wang J and Qian K *et al.* Extremely stretchable strain sensors based on conductive self‒healing dynamic cross-links hydrogels for human-motion detection. *Adv Sci* 2017; **4:** 1600190.
33. Jiang P-P, Qin H and Dai J *et al.* Ultrastretchable and self-healing conductors with double dynamic network for omni-healable capacitive strain sensors. *Nano Lett* 2022; **22:** 1433-42.
34. Chen J, An R, Han L and *et al.* Tough hydrophobic association hydrogels with self-healing and reforming capabilities achieved by polymeric core-shell nanoparticles. *Mater Sci Eng C* 2019; **99:** 460-467.
35. Miao Y, Xu M and Zhang L. Electrochemistry-induced improvements of mechanical strength, self-healing, and interfacial adhesion of hydrogels. *Adv Mater* 2021; **33:** 2102308.
36. Chen C, Wang Y and Zhou T *et al.* Toward strong and tough wood-based hydrogels for sensors. *Biomacromolecules* 2021; **22:** 5204-13.
37. Hua M, Wu S and Ma Y *et al.* Strong tough hydrogels via the synergy of freeze-casting and salting out. *Nature* 2021; **590:** 594-9.
38. Yu W, Cui Y and Han M *et al.* Mussel-inspired chemistry in producing mechanically robust

and bioactive hydrogels as skin dressings. *Mater Today Energy* 2023; **27:** 101272.
39. Dong X, Guo X and Liu Q *et al.* Strong and tough conductive organo‐hydrogels via freeze‐casting assisted solution substitution. *Adv Funct Mater* 2022; **32:** 2203610.
40. Kong W, Wang C and Jia C *et al.* Muscle-inspired highly anisotropic, strong, ion-conductive hydrogels. *Adv Mater* 2018; **30:** 1801934.
41. Yue L, Zhang X and Li W *et al.* Quickly self-healing hydrogel at room temperature with high conductivity synthesized through simple free radical polymerization. *J Appl Polym Sci* 2019; **136:** 47379.
42. Deng Z, Hu T and Lei Q *et al.* Stimuli-responsive conductive nanocomposite hydrogels with high stretchability, self-healing, adhesiveness, and 3D printability for human motion sensing. *ACS Appl Mater Interfaces* 2019; **11:** 6796-808.
43. Stankovich S, Dikin DA and Dommett GHB *et al.* Graphene-based composite materials. *Nature* 2006; **442:** 282-6.
44. Gogurla N, Roy B and Min K *et al.* A skin‐inspired, interactive, and flexible optoelectronic device with hydrated melanin nanoparticles in a protein hydrogel-elastomer hybrid. *Adv Mater Technol* 2020; **5:** 1900936.
45. Han L, Yan L and Wang M *et al.* Transparent, adhesive, and conductive hydrogel for soft bioelectronics based on light-transmitting polydopamine-doped polypyrrole nanofibrils. *Chem Mater* 2018; **30:** 5561-72.
46. Chen Y, Lu K and Song Y *et al.* A skin-inspired stretchable, self-healing and electro-conductive hydrogel with a synergistic triple network for wearable strain sensors applied in human-motion detection. *Nanomaterials* 2019; **9:** 1737.
47. Zhang S, Chen Y and Liu H *et al.* Room‐temperature‐formed PEDOT:PSS hydrogels enable injectable, soft, and healable organic bioelectronics. *Adv Mater* 2020; **32:** 1904752.
48. Lee YY, Kang HY and Gwon SH *et al.* A strain-insensitive stretchable electronic conductor: PEDOT:PSS/acrylamide organogels. *Adv Mater* 2016; **28:** 1636-43.
49. Lu B, Yuk H and Lin S *et al*. Pure PEDOT:PSS hydrogels. *Nat Commun* 2019; **10:** 1043.
50. Del Agua I, Mantione D and Casado N *et al.* Conducting polymer iongels based on PEDOT and guar gum. *ACS Macro Lett* 2017; **6:** 473-8.
51. Chen J, Peng Q and Thundat T *et al.* Stretchable, injectable, and self-healing conductive hydrogel enabled by multiple hydrogen bonding toward wearable electronics. *Chem Mater* 2019; **31:** 4553-63.
52. Ma M, Shang Y and Shen H *et al.* Highly transparent conductive ionohydrogel for all-climate wireless human-motion sensor. *Chem Eng J* 2021; **420:** 129865.
53. Ding Y, Zhang J and Chang L *et al.* Preparation of high-performance ionogels with excellent transparency, good mechanical strength, and high conductivity. *Adv Mater* 2017; **29:** 1704253.
54. Tan Y, Zhang Y and Zhang Y *et al.* Dual cross-linked ion-based temperature-responsive conductive hydrogels with multiple sensors and steady electrocardiogram monitoring. *Chem Mater* 2020; **32:** 7670-8.
55. Zhang X, Cui C and Chen S *et al.* Adhesive ionohydrogels based on ionic liquid/water binary solvents with freezing tolerance for flexible ionotronic devices. *Chem Mater* 2022; **34:** 1065-77.
56. Villa S, Mazzola VM and Santaniello T *et al.* Soft piezoionic/piezoelectric nanocomposites

based on ionogel/$BaTiO_3$ nanoparticles for low frequency and directional discriminative pressure sensing. *ACS Macro Lett* 2019; **8:** 414-420.
57. Liu C, Zhang H J and You X *et al.* Electrically conductive tough gelatin hydrogel. *Adv Electron Mater* 2020; **6:** 2000040.
58. Dai S, Wang S and Yan H *et al.* Stretchable and self-healable hydrogel-based capacitance pressure and strain sensor for electronic skin systems. *Mater Res Express* 2019; **6:** 0850b9.
59. Ren J, Liu Y and Wang Z *et al.* An anti‐swellable hydrogel strain sensor for underwater motion detection. *Adv Funct Mater* 2022; **32:** 2107404.
60. Kim S K, Lee G H and Jeon C *et al.* Bimetallic nanocatalysts immobilized in nanoporous hydrogels for long-term robust continuous glucose monitoring of smart contact lens. *Adv Mater* 2022; **34:** 2110536.
61. Liu H, Wei S and Qiu H *et al.* Supramolecular hydrogel with orthogonally responsive R/G/B fluorophores enables multi‐color switchable biomimetic soft skins. *Adv Funct Mater* 2022; **32:** 2108830.
62. Ding B, Zeng P and Huang Z *et al.* A 2D material-based transparent hydrogel with engineerable interference colours. *Nat Commun* 2022; **13:** 1212.
63. Wang Y, Yu Y and Guo J *et al.* Bio‐inspired stretchable, adhesive, and conductive structural color film for visually flexible electronics. *Adv Funct Mater* 2020; **30:** 2000151.
64. Tsai MS, Shen TL and Wu HM *et al.* Self-powered, self-healed, and shape-adaptive ultraviolet photodetectors. *ACS Appl Mater Interfaces* 2020; **12:** 9755-65.
65. Wu Y, Chen F and Huang N *et al.* Near-infrared light-responsive hybrid hydrogels for the synergistic chemo-photothermal therapy of oral cancer. *Nanoscale* 2021; **13:** 17168-82.
66. Wei Y, Xiang L and Zhu P *et al.* Multifunctional organohydrogel-based ionic skin for capacitance and temperature sensing toward intelligent skin-like devices. *Chem Mater* 2021; **33:** 8623-34.
67. Lin M, Zheng Z and Yang L *et al.* A high‐performance, sensitive, wearable multifunctional sensor based on rubber/CNT for human motion and skin temperature detection. *Adv Mater* 2022; **34:** 2107309.
